# Dynamic reconfiguration of the default mode network during narrative comprehension

**DOI:** 10.1038/ncomms12141

**Published:** 2016-07-18

**Authors:** Erez Simony, Christopher J Honey, Janice Chen, Olga Lositsky, Yaara Yeshurun, Ami Wiesel, Uri Hasson

**Affiliations:** 1 Department of Psychology, Princeton University, Princeton, New Jersey 08540-1010, USA; 2 Princeton Neuroscience Institute, Princeton University, Princeton, New Jersey 08540-1010, USA; 3 Department of Psychology, University of Toronto, Toronto, Ontario, Canada M5S 3G3; 4 School of Computer Science and Engineering, Faculty of Science, The Hebrew University of Jerusalem, 9190416 Jerusalem, Israel

## Abstract

Does the default mode network (DMN) reconfigure to encode information about the changing environment? This question has proven difficult, because patterns of functional connectivity reflect a mixture of stimulus-induced neural processes, intrinsic neural processes and non-neuronal noise. Here we introduce inter-subject functional correlation (ISFC), which isolates stimulus-dependent inter-regional correlations between brains exposed to the same stimulus. During fMRI, we had subjects listen to a real-life auditory narrative and to temporally scrambled versions of the narrative. We used ISFC to isolate correlation patterns within the DMN that were locked to the processing of each narrative segment and specific to its meaning within the narrative context. The momentary configurations of DMN ISFC were highly replicable across groups. Moreover, DMN coupling strength predicted memory of narrative segments. Thus, ISFC opens new avenues for linking brain network dynamics to stimulus features and behaviour.

## Abstract (web summary)

Default mode network (DMN) is strongly modulated by idiosyncratic internal processes, but its involvement in processing external stimuli is unclear. Here, Simony and colleagues use an inter-subject functional correlation approach to extract DMN states that track stimulus features and behaviour.

## Graphical abstract

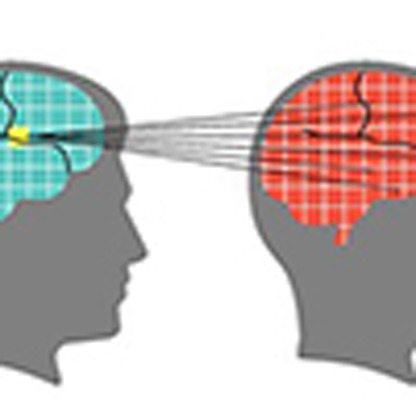

In everyday settings, such as watching a movie or listening to a lecture, it is necessary to accumulate and integrate information over many minutes. We have previously identified a set of high-order brain areas, including the temporal parietal junction, angular gyrus, precuneus, posterior cingulate cortex (PCC), medial prefrontal cortex and temporal pole, which can accumulate and integrate information over minutes-long timescales^1^,^2^,^3^. These areas with long processing timescales appear to coincide with members of the default mode network (DMN), a network whose function is only partly understood despite intense scrutiny over the past decade^4^,^5^.

There is a longstanding puzzle concerning the role of the DMN in active processing of information from the world. Functional connectivity (FC) between DMN regions continually fluctuates during externally focused tasks, during rest and even during sleep^6^,^7^,^8^,^9^. Diverse network states are observed from minute to minute, varying around an architecture that is moderately stable when averaged over tens of minutes^10^. On the basis of studies that have employed pre- and post-task FC patterns^11^,^12^ and short event-related designs^4^,^13^,^14^,^15^, DMN function has been linked to the recollection of past episodes and the simulation of future episodes^14^, theory of mind^16^ and tasks involving social content^17^.

But do the varying inter-regional correlations of the DMN reflect active processing of external information in the present moment? For example, might denser or sparser DMN connectivity reflect different kinds of episodic representations of the present, with distinct consequences for memory of each episode? And how could such an active role for the DMN be reconciled with the longstanding observation that DMN topography is only very weakly modulated by the presence or absence of external stimulation^9^,^18^,^19^,^20^?

To better characterize the dynamic changes in DMN correlation patterns that are locked to the processing of external stimuli, we introduce a novel method termed inter-subject functional correlation (ISFC), in which inter-region correlations are calculated between different brains exposed to the same continuous real-life stimulus (an auditory narrative). We demonstrate that the network correlation patterns detected using standard FC analysis primarily reflect intrinsic blood-oxygen-level dependent (BOLD) fluctuations within each brain. In contrast, the ISFC method increases the signal-to-noise ratio (SNR) in detecting stimulus-induced inter-regional correlation. The improvement in SNR arises from filtering out intrinsic neural dynamics (for example, responses arising from intrinsic cognitive processes unrelated to ongoing stimulus processing), as well as non-neuronal artifacts (for example, respiratory rate; motion) that can influence network correlation patterns *within* a brain but are not correlated *across* brains.

The ISFC approach uncovered two novel functional characteristics of DMN correlation patterns. First, DMN correlation patterns were less reliable when the story was scrambled at the paragraph level, and even less so when the story was scrambled at the word level. This suggests that DMN correlations were modulated by the coherence of the stimulus' temporal structure over minutes of time, consistent with the notion that a long-timescale history-dependent representation is maintained within the DMN. Second, the pattern of DMN correlation reconfigured in a manner that was (i) specific to individual segments of the story and (ii) highly replicable across independent groups of subjects. Thus, the inter-subject functional correlation approach provides a powerful new view on large-scale neural dynamics.

## Results

### Study design

To characterize stimulus-induced DMN correlation patterns, we collected BOLD responses during narrative-driven and resting state conditions. Subjects listened to a real-life story, as well as to word scramble and paragraph scramble versions of the story. We also collected 10 min of eyes-open resting data. For an additional nine subjects, respiration and heart rate (HR) were measured during scanning of the intact story and resting state conditions.

The results below include four main sections: (i) we present the ISFC method together with a statistical model explaining the gain in SNR that it provides; (ii) we demonstrate that ISFC patterns in the DMN track the level of coherence of the narrative; (iii) we show that we can use ISFC in the DMN to decode, within single subjects, the specific segment of the narrative that is being presented; (iv) we show how ISFC reveals intricate and yet highly reliable inter-network dynamics, in particular between the DMN and task-positive regions.

### BOLD signal decomposition and the rationale behind ISFC

**Figure 1:**
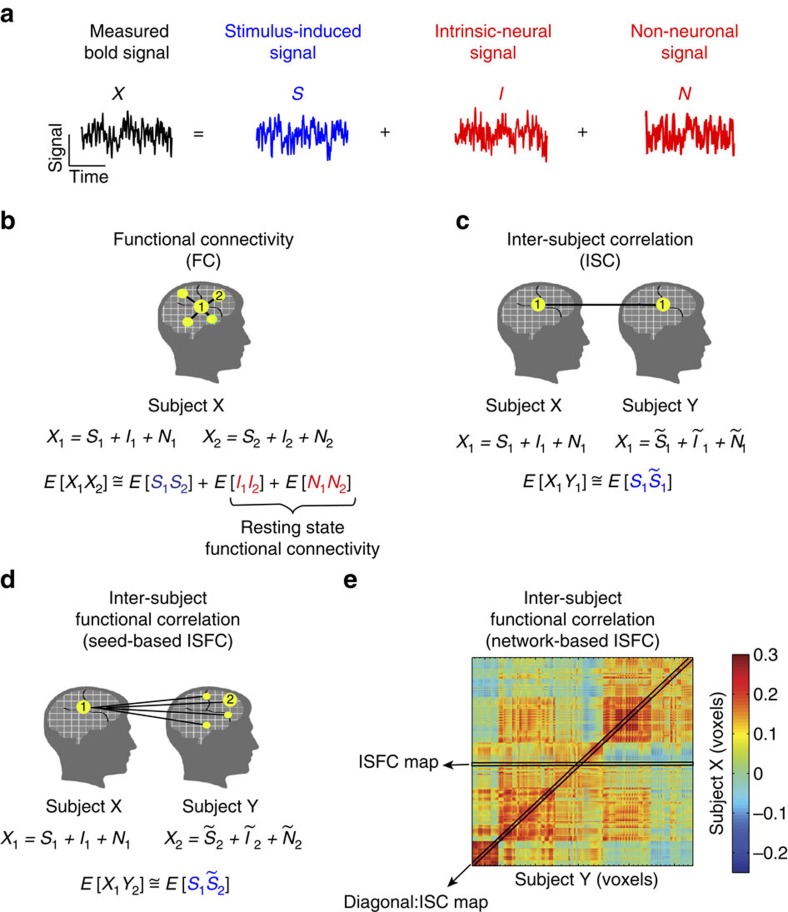
Inter-subject functional correlation (ISFC) method. (**a**) During task processing the measured BOLD signal can be decomposed into stimulus-induced signal (blue), intrinsic neural signal (spontaneous fluctuations) and non-neuronal signal (for example, physiological noise) (red). (**b**) Seed-based functional connectivity (FC) is the Pearson correlation between a time course extracted from a seed region (1) in subject X, and all other regions in the same subject, for example, region (2). This can be estimated as the sum of stimulus-induced correlations (blue) and intrinsic neural correlations (red), which are difficult to separate. (**c**) Point-to-point inter-subject correlations (ISC): stimulus-induced correlation (blue) between time courses from the same region (for example, region 1) across subjects X and Y. ISC reveals stimulus-induced within-region correlations that are shared across subjects. (**d**) Seed-based ISFC is the Pearson correlation between a time course extracted from one region in subject X and all other regions in subject Y (for example, Region 1 in subject X versus region 2 in subject Y). (**e**) Network-based ISFC are the Pearson correlations between a network of brain regions in subject X and a network of brain regions in subject Y. This correlation matrix computed across brains (the diagonal represents ISC) filters out intrinsic and non-neuronal correlations and highlights stimulus-induced inter-regional correlations that are shared across subjects. For a statistical model and its analytical solution see Supplementary Note 1.

We model the measured BOLD signal in each voxel as a sum of three components (Fig. 1a): stimulus-induced signal (S), intrinsic neural signal (I) and non-neuronal (for example, physiological) noise signal (N)^21^,^22^,^23^,^24^,^25^. The stimulus-induced signal (S) is defined as the neural responses that are time-locked to the processing of the external stimulus. The intrinsic neural signal (I) is defined as the neural responses that are not reliably related to the processing of external stimuli. Such intrinsic neural signals should be uncorrelated across subjects. Similarly, most studies assume (and we validate this below for our setting, Supplementary Fig. 1) that non-neuronal artifacts (N), such as head motion, respiration and HR are also uncorrelated across subjects.

In a task setting, the pattern of correlations within each individual, as computed by the FC method, will be influenced by each of the three components (Fig. 1b). In contrast, inter-subject correlation (ISC)^26^ captures the stimulus-induced correlation across subjects within a given region by correlating time courses from the same region across subjects (Fig. 1c).

### ISFC and ISC

In previous studies, we used ISC to quantify the reliability of stimulus-driven responses within each brain area (Fig. 1c); here we introduce ISFC, which provides inter-regional covariance information unavailable from ISC. The ISFC approach extends ISC by characterizing the correlations between (a) response time courses for one seed region in one subject, and (b) the response time courses in all voxels of all other subjects (Fig. 1d, Methods). Going beyond this seed-based ISFC, one can also calculate the inter-regional correlation matrix across all pairs of voxels across brains (Fig. 1e). The diagonal of this voxel-wise correlation matrix is identical to ISC. A single seed-based ISFC map for a particular voxel (Fig. 1d) is depicted by a single row or column of the correlation matrix (Fig. 1e). ISFC uncovers novel information about inter-regional stimulus-induced correlation patterns that is not captured by ISC, because the off-diagonal elements of the correlation matrix are not determined by the diagonal elements of the matrix. Furthermore, by calculating ISFC over a short (45–90 s) sliding window, it is possible to isolate highly reliable stimulus-induced changes in the correlation patterns across nodes of the DMN. In the Supplementary Note section, we provide a detailed statistical model with a formal analytical solution to explain the SNR gain in detecting shared stimulus-induced network correlation patterns by using ISFC over FC. Furthermore, throughout the paper we empirically quantify, using a classification approach, the gain in information extracted about the stimulus by using ISFC instead of FC.

### ISFC filters out intrinsic correlations and noise

**Figure 2:**
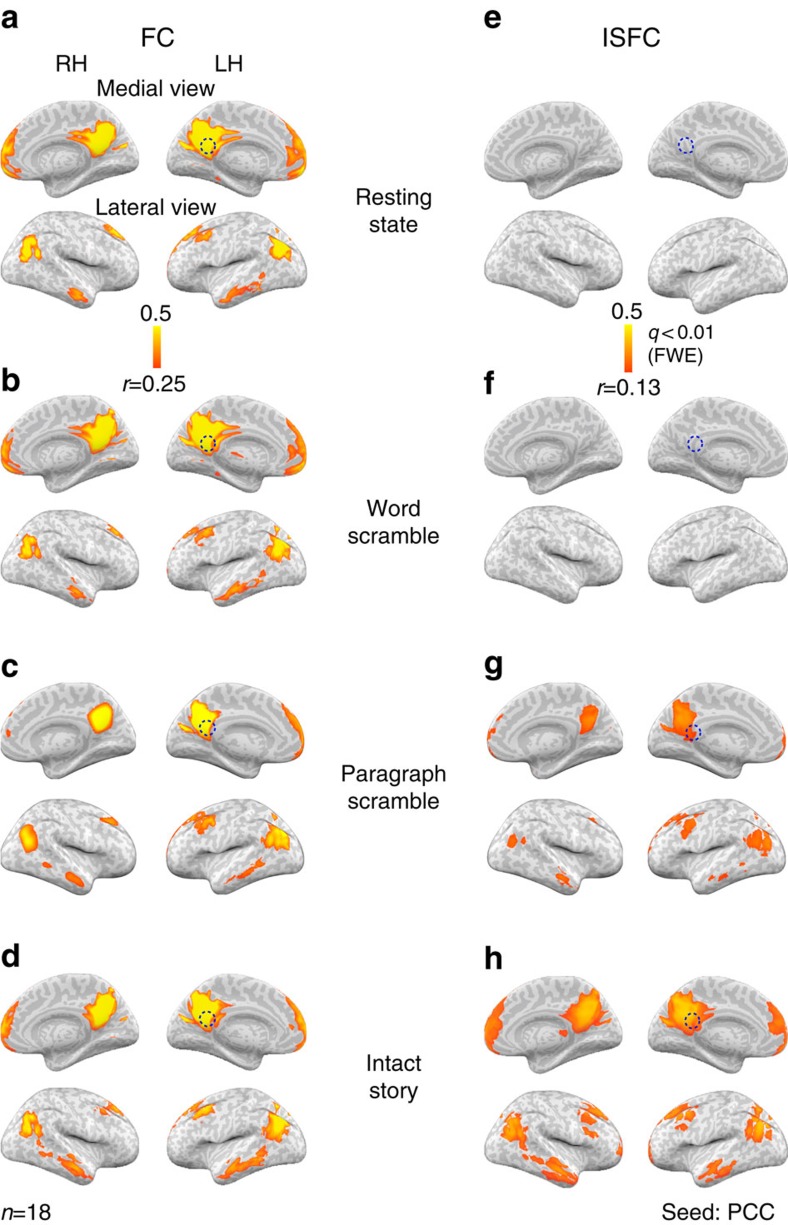
Seed-based ISFC reveals stimulus-induced correlations in the default mode network (DMN) during narrative comprehension. (**a**–**d**) Average functional connectivity (FC) maps across 18 subjects between the posterior cingulate cortex (PCC) seed (dashed circle) and the entire brain reveals the same DMN during (**a**) resting state, (**b**) word scramble, (**c**) paragraph scramble and (**d**) intact story conditions (*r*>0.25, nonparametric family-wise error, FWE, correction *q*<0.01). (**e**–**h**) Inter-subject functional correlation (ISFC) between the PCC seed (dashed circle) and the entire brain across 18 subjects reveals no significant stimulus-induced correlations (*r*<0.1, *q*>0.1) in the DMN during resting state (**e**) or word scramble (**f**). In contrast, seed-based ISFC reveals significant stimulus-induced correlations in the DMN during paragraph scramble (**g**) and maximum correlations during intact story (**h**) (*r*>0.13, FWE *q*<0.01). See also Supplementary Fig. 2. RH, right hemisphere; LH, left hemisphere.

Intrinsic signals (I) and noise signals (N) during rest cannot induce inter-regional correlations across subjects. In the absence of an external stimulus or explicit task, the BOLD signal is dominated by intrinsic fluctuations^27^, which can be used to map brain networks via standard FC methods. Following standard approaches^28^,^29^, we defined the DMN (Fig. 2a) by computing the FC map for a seed in the PCC in each subject (*n*=18) during rest, and then averaging the FC maps across all subjects. The resulting network comprised the precuneus/PCC, middle frontal gyrus, medial prefrontal cortex, inferior parietal lobule and middle temporal gyrus. Statistical significance for all ISFC and FC maps in the paper was assessed using a permutation procedure based on phase-randomized surrogate data. Family-wise error rate (FWER) was controlled across the brain using a max-based resampling procedure (see Methods). While standard FC analysis revealed the DMN, no significant correlations were found across subjects (that is, there was no ISFC) during rest for the same PCC seed (Fig. 2e, empty map). Thus, intrinsic neural processes (I) and non-neuronal noise sources (N) did not elicit ISFC across subjects. This is in contrast to FC analyses, which reflect a combination of stimulus-driven responses, intrinsic dynamics and noise sources such as HR^30^,^31^, respiration^32^,^33^ and motion^34^,^35^. We empirically demonstrate the presence of these noise sources in FC analyses, and their absence in ISFC analyses, both during rest and the intact story, in Supplementary Fig. 1.

### Inter-subject alignment of the DMN

We next compared the patterns of FC and ISFC in the DMN across four distinct conditions (intact story, paragraph scramble, word scramble and resting state). The FC and ISFC analyses were performed using the same PCC seed. We observed little variation in the FC across the rest condition (no stimulus, Fig. 2a), the word scramble condition (Fig. 2b), the paragraph scramble condition (Fig. 2c) and the intact story condition (Fig. 2d). Thus, the DMN structure defined using the standard FC analysis was similar across conditions and relatively unaffected by the presence or absence of external stimuli^9^. By contrast, when computing ISFC using the same seed region in the same data set, correlations varied substantially across the four experimental conditions: the same DMN as was seen in FC was observed in ISFC for the paragraph scramble and intact story conditions (Fig. 2g,h), but the ISFC maps for the resting (Fig. 2e) and word scramble conditions (Fig. 2f) were empty. Thus, the ISFC method exposed a robust stimulus-induced alignment of DMN nodes across subjects, which was selective to the processing of a coherent narrative. In contrast, the standard FC maps were dominated by stimulus-independent components, which did not distinguish between different levels of narrative coherence. This advantage for ISFC over standard FC did not depend on the choice of statistical threshold applied to the correlation maps (see Supplementary Fig. 2).

### DMN is locked to the high-level properties of the stimuli

**Figure 3:**
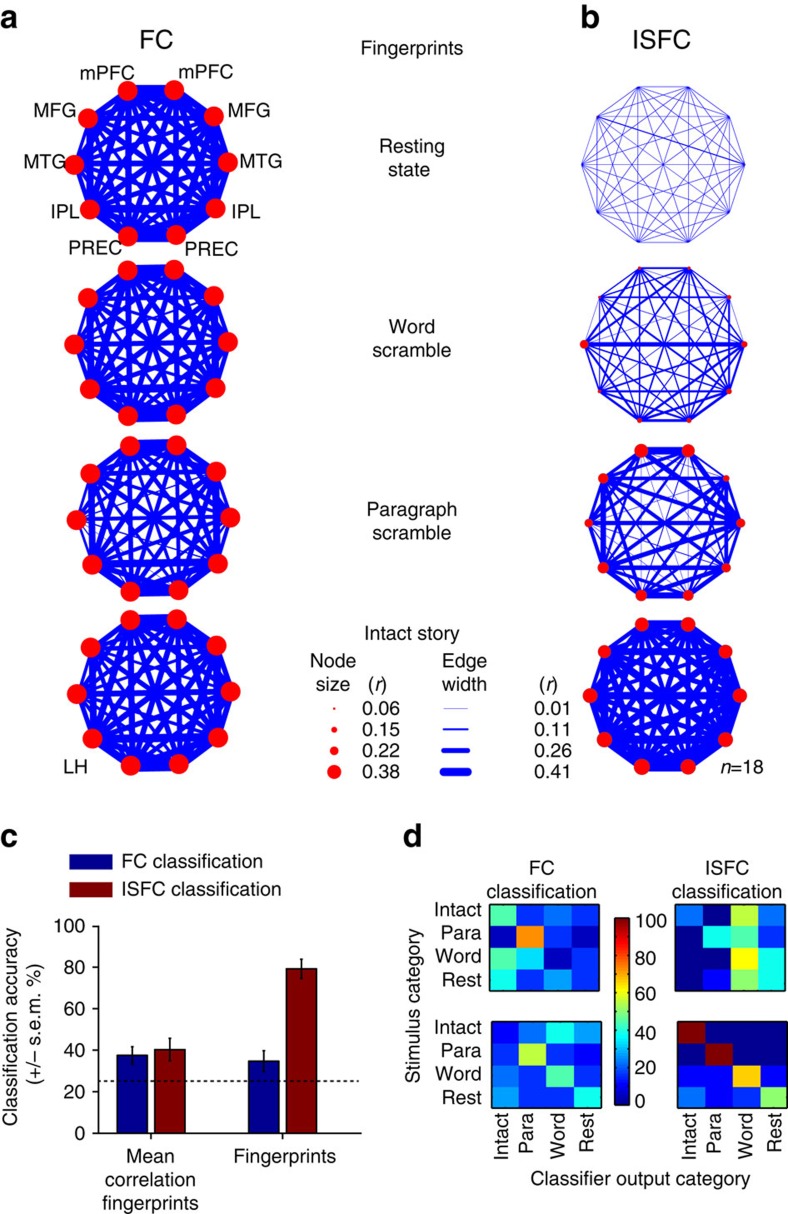
Network-based ISFC patterns are locked to the temporal coherence levels of the story. (**a**) Network-based FC patterns of the DMN across four conditions (resting state, word scramble, paragraph scramble, intact story). Line width represents the pairwise correlation between different nodes (edge width) in DMN, while circle size represents the correlation between the same nodes (node size). In FC, the correlation of a node with itself is always 1, and the size of the red circles is, therefore, uninformative and fixed. In the ISFC framework, however, the size of the node denotes the correlation of the node across subjects (that is, ISC), so the node size can vary. (**b**) Network-based ISFC patterns within the DMN across the four conditions (resting state, word scramble, paragraph scramble, intact story). (**c**) Across-subject classification of task condition using ISFC and FC (four possible conditions, chance=25%). Classification was performed either using the mean correlation across all edges (left bars) or using the full ‘fingerprint' of the correlation pattern, which is a 10 × 10 matrix of pairwise correlations between brain regions (right bars). (**d**) Confusion matrices of FC (left) and ISFC (right) classification across the four conditions. Features for classification were the entire fingerprints (bottom) or only the mean of the fingerprint (top), used to decode condition (level of scrambling).

To examine the variation in DMN correlation patterns across stimuli between all regions of the DMN (as opposed to only examining correlations with the PCC seed), we next applied FC and ISFC analyses to a set of bilateral DMN regions of interest (ROIs). We defined the DMN network nodes in a standard manner by seeding the PCC, computing a resting state FC map, and isolating the five major DMN nodes in each hemisphere^4^. Using the resulting ten DMN ROIs (Fig. 2a), we calculated the network-based FC (Fig. 3a) and network-based ISFC (Fig. 3b) for each of four conditions: rest, word scramble, paragraph scramble and intact story conditions (see Methods). The thickness of the blue lines (edges) represents the magnitude of positive correlation between two different ROIs within subjects (FC) or across subjects (ISFC). Each node of the network is marked by a red circle and represents an ROI.

Averaged FC network correlations in the DMN were largely invariant across experimental conditions (Fig. 3a), whereas ISFC patterns distinguished between them. The averaged ISFC network correlations increased with the temporal coherence of the stimulus, from the word scramble condition to the intact story condition (Fig. 3b). The ISFC values in the rest data were again low (−0.1<*r*<0.1) and statistically indistinguishable from zero (threshold, R*=0.1 at *q*>0.01 corrected for FWER). A simulation based on our statistical model (Supplementary Note 1) confirmed that ISFC provides a dramatic increase in SNR for detecting the stimulus-induced correlation patterns compared with standard FC analyses (see Supplementary Fig. 3).

### Across-subject classification of stimulus type

Next, we trained a classifier to quantify the improvement in discriminating between the four experimental conditions by using ISFC over FC. Classification was performed separately using ISFC and using FC (see Supplementary Note 2). Classification accuracy between four conditions (Intact versus paragraph scramble versus word scramble versus rest) was significantly better with ISFC than FC when using the full correlation fingerprint (Fig. 3c, 37% versus 80%). This advantage for ISFC persisted (35% versus 40%) even when only the mean correlation across all edges served as input to the classifier, rather than the full fingerprint of all correlations. The confusion matrices across conditions present more detailed information about classification match and mismatch (Fig. 3d). Overall, we observed that ISFC patterns within the DMN enabled superior decoding of stimulus condition compared with using FC patterns. Similar improvement in classification performance was observed when we measured the stimulus-induced inter-regional correlation patterns between the DMN and all other functional networks at the single voxel level (See Supplementary Fig. 10). The classification advantage of ISFC over FC was also observed when classifying conditions in a standard block-design paradigm (Supplementary Fig. 5).

### ISFC reveals reliable dynamics of DMN correlation patterns

ISFC allowed us to characterize the dynamics of stimulus-induced correlations within the DMN over the course of the story, and with far greater SNR than a standard FC analysis. To measure changes in stimulus-induced correlation patterns over time, we calculated the ISFC within a sliding window of 90 s (60 TRs (repetition time)). At each time point*, t*, we calculated the pairwise correlations across all nodes of the DMN (network-based ISFC) over the window interval (*t*,*t*+90). Windows were shifted by 1.5 s (1 TR) along the story. First, we extracted the mean correlations across all network edges within each window, to obtain a global measure of the network state. Next, we examined the specific pattern of correlations across different nodes of the DMN within each window.

**Figure 4:**
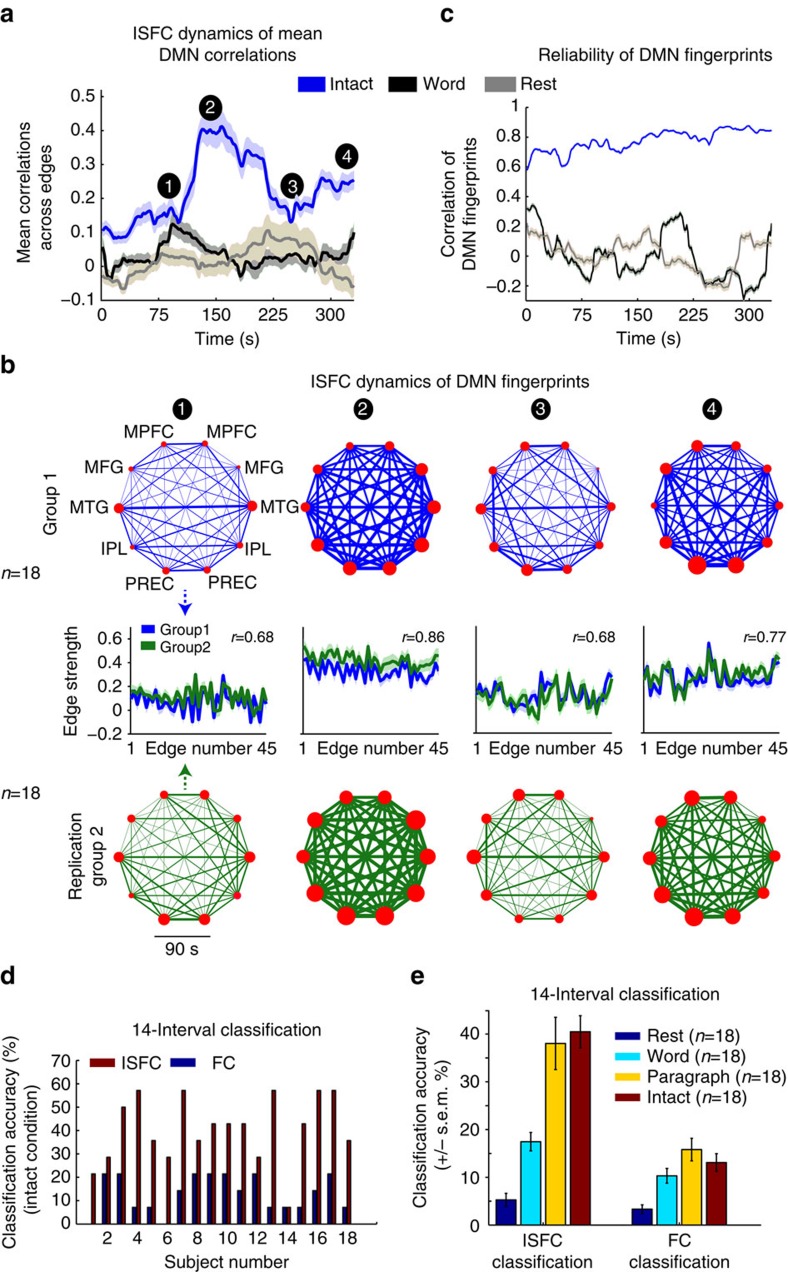
ISFC dynamics of the DMN correlation fingerprints during the intact story are distinct and replicable over time. (**a**) Mean ISFC of all edges in the DMN computed over time in 90 s sliding windows (window at time *t* is data from (*t*, *t*+90 s) with a step-size of 1.5 s between windows). The ISFC across 18 subjects is shown for the intact story (blue), word scramble (black) and rest (grey) conditions. (**b**) DMN correlation patterns (DMN fingerprints) in four time intervals (1, 2, 3, 4) during the intact story condition in group 1 (18 subjects, blue) and in replication group 2 (18 subjects, green); middle: the DMN pairwise edge correlations (for example, right mPFC-right MTG) across the two groups. For the labels of all 45 edges, see Supplementary Table 1. (**c**) High ISFC pattern reliability in the DMN over time (sliding window of 90 s, step size 1.5 s) across two groups of 18 subjects shown for the intact story (blue), word scramble (black) and rest (grey) conditions. See also Supplementary Figs 6 and 7. (**d**) Across-subject classification of DMN fingerprints, using ISFC and FC fingerprints, over 14 non-overlapping intervals (14 × 30 s) at the level of single subjects (chance level, 7%). (**e**) Mean classification accuracy using ISFC and FC fingerprints over 14 non-overlapping intervals, within four conditions (chance level 7%). Eighteen subjects were used for each condition (resting state, word scramble, paragraph scramble, intact story).

The mean level of stimulus-induced correlations in the DMN was modulated throughout the story (Fig. 4a, blue). Mean ISFC within the DMN was low (*∼r*=0.1) in the first 2 min of the story, then increased to a peak of *r*=0.4 around 150 s, and then decreased and increased again towards the end of the story. The changes in the mean ISFC over time in the DMN were reproduced across two independent groups, using 45 s (30 TRs) and 90 s (60 TRs) sliding windows (Supplementary Fig. 6a,b). This alignment of the DMN network dynamics across subjects was not observed during the word scramble condition (Fig. 4a, black) or in the resting state group (Fig. 4a, grey). In contrast, using FC, the mean correlation of the DMN edges was high for all conditions. The FC analysis in the intact condition revealed similar modulations of FC over time to those revealed by ISFC, but they were much smaller and noisier, because the stimulus-induced component was not isolated (Supplementary Fig. 7a). Overall, these results suggest that the intact story elicited reliable changes in DMN correlations over time, and that these stimulus-induced correlations are induced by high-level features of the story (for example, the narrative structure unfolding over time) rather than low-level aspects of the story (for example, lexical processing), which were preserved in the scrambled words condition.

### Replication of ISFC patterns in independent subjects

To quantify the replicability of the stimulus-specific network correlation patterns estimated using ISFC, we recomputed ISFC patterns of the DMN within the two groups of 18 subjects in the intact story and rest conditions. The ISFC patterns in the DMN during rest between groups were essentially random (*r*=0.01, *P*>0.05, Supplementary Fig. 4b). However, during the intact story, the ISFC patterns were highly reproducible across group 1 and group 2 (*r*=0.88, *P*<0.01 for ISFC, Supplementary Fig. 4c). Thus, the stimulus-induced network configuration revealed by ISFC during narrative processing replicates across independent groups of subjects. Finally, we quantified the number of subjects needed to extract reliable, stimulus-locked patterns in the DMN using the ISFC method (Supplementary Fig. 4d). While we observed non-replicable ISFC patterns during rest (grey curve), we observed replicable ISFC patterns in the DMN during the intact condition (blue curve) even with only two subjects. Increasing the number of subjects led to an increase in ISFC pattern replicability, which approached *r*=0.82 with 18 subjects per group. Reliable changes in the DMN correlations over time were also found using another audio-visual movie (see Supplementary Fig. 8). Importantly, the changes in the mean ISFC over time during the movie had a unique temporal trajectory, distinct from the temporal trajectory observed for the auditory story.

### Dynamics of ISFC fingerprints

Was the mean magnitude of network correlations simply being scaled upward and downward over the course of the narrative, or were DMN ISFC patterns reconfigured into distinct patterns for each story segment? To answer this question, we inspected the network state within four intervals of the story (Fig. 4b). Two intervals exhibited low mean ISFC (Fig. 4a, labels 1 and 3) and two exhibited higher mean ISFC (Fig. 4a, labels 2 and 4). The DMN correlation patterns (‘fingerprints') for each interval are plotted in Fig. 4b, with ISFC patterns for one group of 18 subjects in blue, and a replication group of 18 subjects in green.

The ISFC patterns were specific for different moments in time and also highly reproducible across two independent groups of subjects. Interestingly, the reproducibility of ISFC patterns was observed both when the mean ISFC across all nodes was high and when it was low: that is, reproducibility was high when the correlation matrix was sparse (intervals 1 and 3), as well as when it was dense (intervals 2 and 4). Moreover, the similarity between these four correlation patterns was low (Supplementary Fig. 7e), indicating a specific ISFC configuration for each of the story's segments. By contrast, when we applied a standard within-brain FC approach to the same data sets, the FC configurations were not selective for different parts of the story, evincing highly similar structure over time (Supplementary Fig. 7c,d). Thus, by extracting the stimulus-induced component of the neural dynamics using ISFC, we revealed robust and reproducible network states that were diagnostic for specific intervals of the stimulus.

Finally, we extended the previous analysis from four intervals to an analysis of all 90 s-intervals in the stimulus, by repeatedly and randomly assigning 18 subjects to each group, for each condition, and calculating the correlations between ISFC patterns across groups (see Methods). We confirmed that the ISFC correlation patterns are highly reliable across the groups throughout all intervals of the intact story condition (Fig. 4c, blue line). By contrast, the ISFC patterns in the word scramble and the rest conditions show little consistency across the original and replication subject groups (Fig. 4c, black and grey lines). This more temporally fine-grained analysis also reproduced the pattern of FC results, in which average FC patterns showed little variation over time and conditions and were less selective for specific moments in the stimulus (Supplementary Fig. 7b).

### Across-subject classification of story intervals

The unique ISFC patterns observed for each segment of the story in the DMN can be used to classify the story's temporal intervals. Furthermore, while ISFC is computed by correlating response time courses across brains, it also uncovers stimulus-locked correlation patterns within each individual. To demonstrate this, we conducted classification at the individual subject level (Fig. 4d) as well as on the average group level (Fig. 4e). In this analysis, we divided the story into 14 non-overlapping intervals of 20 TRs (30 s), and compared ISFC classification performance to FC classification (see Supplementary Note 2). Our results revealed an increase in the accuracy of decoding single intervals in the story within each subject when using ISFC compared with FC (Fig. 4d). Given that chance level is low (7.1%) the ISFC classification performance at the single subject level is notable. For the intact story, at the group level (averaged across subjects), ISFC enabled interval decoding accuracy of 42±3.3% (*P*<0.01), while using FC the performance drops to 13±1.8% (Fig. 4e). Congruent with the observation that stimulus-locked responses in the DMN diminished for the scrambled conditions, we observed a decline in classifier performance when trying to predict interval identity using the scrambled versions of the story (word scramble, paragraph scramble and rest), as depicted in Fig. 4e. This again attests to the specificity of the stimulus-locked correlations detected using ISFC analysis.

### ISFC in the DMN depends on minutes of prior information

**Figure 5:**
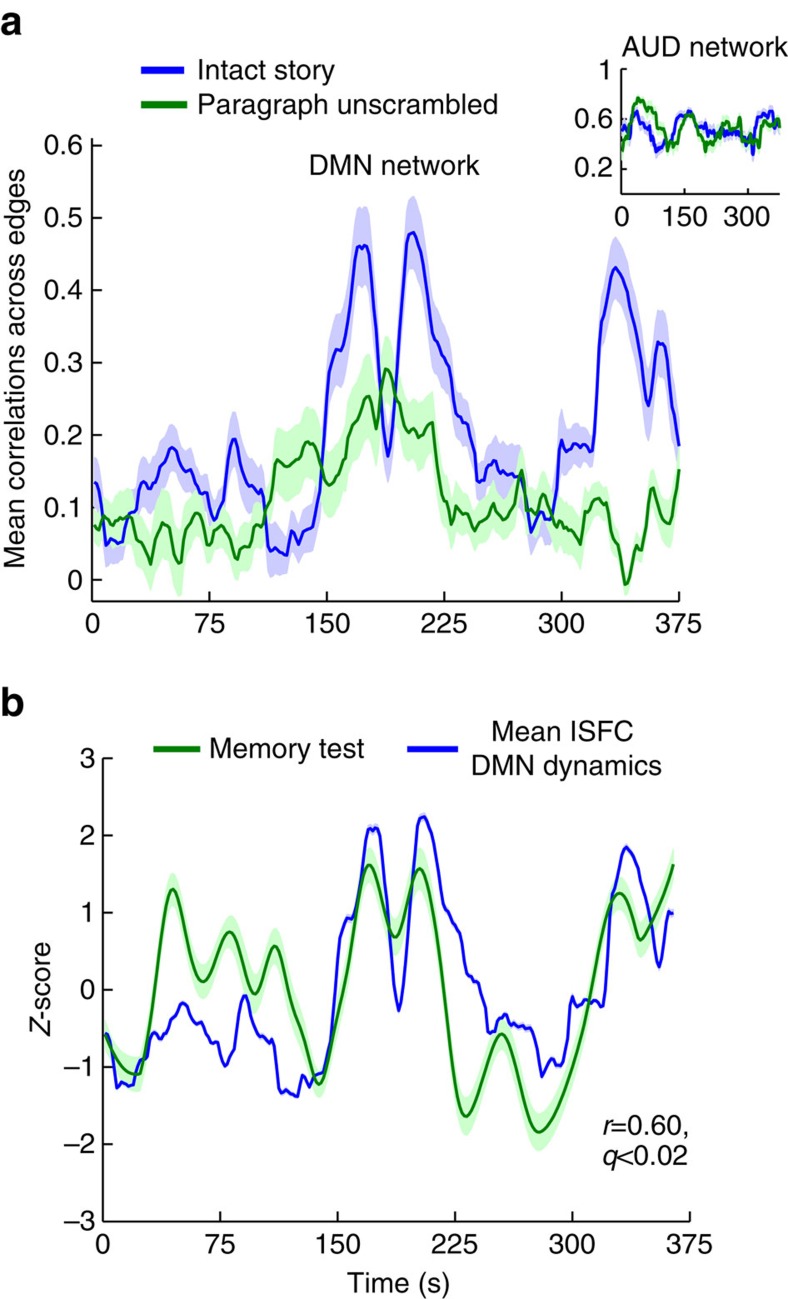
DMN dynamics correlate with recall for story elements. (**a**) Mean ISFC of all edges in the DMN (and auditory cortex, small inset) computed in 45-second sliding windows during intact story (blue) and paragraph scramble conditions (green). Note that the paragraphs were reordered to match the order of presentation in the intact story, and ISFC is shown in the reordered form for comparison with the Intact story. While ISFC dynamics were unaffected by paragraph-ordering in the auditory system, the ISFC dynamics in the DMN were strongly modulated by the same manipulation. (**b**) The mean ISFC in the DMN for each segment of the story was correlated with recall of that segment of the narrative (*r*=0.6, *q*<0.02).

Are the changes in DMN ISFC over the course of the narrative driven by transient features of the story (that is, a history-independent effect) or does the mean ISFC pattern depend on the previous history and context of each moment within the narrative? To answer this question, we reordered the response time courses in the paragraph scramble condition to match the intact story condition. We then compared the time courses of mean ISFC magnitudes across the two conditions (that is, the intact story and paragraph scramble). We performed this comparison both within the DMN and in the auditory network, using a sliding window of 30 TRs (Fig. 5a). In the auditory network, mean ISFC was similar for each paragraph, regardless of whether that paragraph had been presented in the scrambled context or in the intact context (Fig. 5a inset). In contrast, in the DMN, the ISFC time course was qualitatively different when the order of the paragraphs had been scrambled (Fig. 5a), suggesting that the changes in inter-regional correlations in the DMN were indeed history-dependent. This result supports and extends previous findings using ISC^2^.

Given the prior association between DMN function and episodic memory^14^,^36^, we investigated whether the history-dependent ISFC strength in DMN regions was related to successful encoding of the narrative. We ran a detailed post-listening memory test (see Methods) in a separate group of 30 subjects, assessing the accuracy of recollecting story details from 40 intervals of the narrative (Fig. 5b). Subsequent recall accuracy for events from an interval was highly correlated with the degree of stimulus-induced correlations in the DMN (mean ISFC, red line, *r*=0.6, *q*(FWER) <0.02) during that interval. This result suggests that the dynamic reconfiguration of stimulus-induced DMN correlations as the story unfolded was related to the encoding of narrative information in long-term memory.

### ISFC reveals transient negative correlations between networks

**Figure 6:**
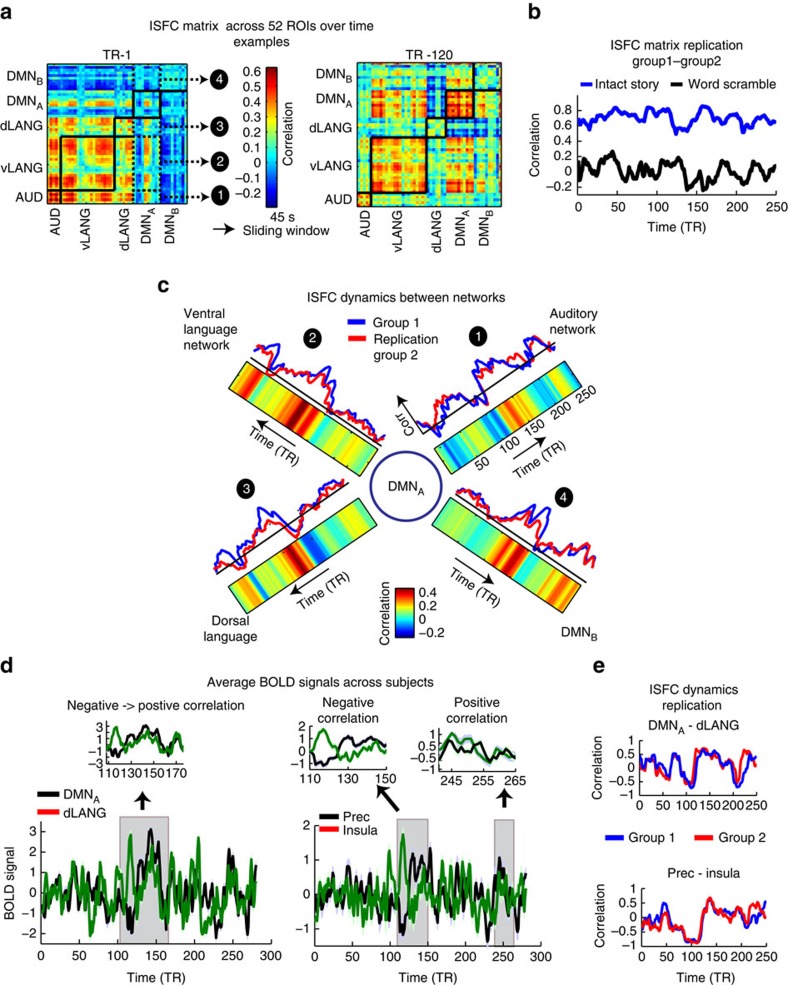
ISFC of the DMN with language areas reveals reliable but transient periods of negative and positive correlations. (**a**) Example correlation matrices derived by computing ISFC over two intervals of 45 s each. Pairwise correlations were calculated between 52 ROIs across five networks. The mean ISFC over the sub-matrices 1–4 represents the interaction between the DMN_A_ and other networks. (**b**) Replication of ISFC correlation matrices across two groups, each of 18 subjects, during intact and word scramble conditions. (**c**) Interactions between the DMN_A_ and dLANG, vLANG, DMN_B_ and auditory networks. ISFC dynamics between networks are replicable across two groups, and transient anticorrelation epochs are seen between the DMN_A_ and the dLANG and auditory networks. (**d**) Average BOLD signal across subjects in the precuneus and the anterior insula exhibit both significant positive and negative correlations over short intervals at different times in the story. (**e**) Replicable dynamics between DMN_A_ and dorsal language network, and between the precuneus and the insula, across two groups, each of 18 subjects, during intact story condition.

Previous studies using FC have reported that the resting brain exhibits intrinsic negative correlations between the DMN (task-negative regions) and the dorsal attention network (task-positive regions)^37^. However, it is still unclear whether these negative correlations reflect relative differences in response patterns that are amplified by regressing out a common global signal, or whether they reflect a true anticorrelation in the responses across different functional networks^38^,^39^. In addition, electrophysiology work has shown that anticorrelations between the DMN and task-positive networks were observed only about 20% of the time, suggesting a more complex inter-relationship that may change over time^40^. In Fig. 6, we calculated ISFC between the DMN_A_ network and four other networks (auditory network, dorsal language network, ventral language network and DMN_B_). Figure 6a presents examples of region-wise correlation matrices (52 × 52 nodes) across all five networks in 45-second windows. Figure 6c presents the average ISFCs across pairs of networks over time, as calculated by the mean of all pairwise correlations across networks. We observed reliable transient changes in the correlation patterns from positive (maximum *r*=0.4) to negative (minimum *r*=−0.22) between the DMN and the dorsal language system, as well as between the DMN and auditory areas. The fluctuations in the correlation patterns can also be seen by inspecting the average BOLD time courses across subjects in the DMN_A_ and dorsal language network (Fig. 6d, left panel). The fluctuations were also seen at the level of single nodes. For example, we observed fluctuations in the sign of the correlation, from positive to negative, between the precuneus and insula (Fig. 6d, right panel). Finally, changes in the correlation patterns were seen at the level of region-wise and voxel-wise correlation matrices (see the two examples of region-wise correlation matrices (52 × 52 nodes) at different time windows in Fig. 6a and see a plot of the changes in the voxel-wise ISFC patterns (8,128 × 8,128 voxels) overall windows in Supplementary Movie 1). Crucially, we replicated all of the above results across two independent groups of subjects, at the average-network level (Fig. 6e, upper, *r*=0.72), the average single-node level (Fig. 6e, Bottom, *r*=0.85), and the region-wise correlation matrix level (Fig. 6b, *r*=0.7 intact story, *r*=0.007 word scramble). Thus, ISFC uncovered reliable changes in connectivity across networks over the course of the intact story, with various regions and networks working in unison at specific times during the narrative, while displaying anticorrelated responses at other times.

## Discussion

To study the DMN's functional properties, one needs to investigate its responses while it processes information from the environment, that is, during task performance. However, this is a challenge for standard FC analyses, which reflect a mixture of stimulus-induced correlations, intrinsic neural correlations and non-neuronal artificial correlations within each subject (Fig. 2 and Supplementary Fig. 1). Here we introduce an approach (ISFC) for isolating stimulus-induced correlations, taking advantage of the fact that intrinsic and non-neuronal artificial signals are uncorrelated across subjects. Using standard FC analyses, averaged DMN correlation patterns were similar across all conditions (resting state, and listening to intact, paragraph scramble and word scramble versions of a real-life story). These FC results are consistent with the notion that the DMN is an ‘intrinsic' network that exhibits only small task-related modulations. In contrast, the ISFC approach uncovered a different pattern (Fig. 3): ISFC patterns in the DMN varied robustly across stimuli, exhibiting greater network correlations as the temporal coherence of the stimulus increased. As opposed to the relatively non-selective FC patterns, ISFC patterns within the DMN changed in a reliable manner as the story unfolded, enabling us to classify different phases of narrative processing (Fig. 4). Importantly, stimulus-induced dynamics in the DMN configurations were not driven by low-level stimulus properties: they changed as a function of the coherence of the narrative (Fig. 5a) and they could be used to predict the memorability of each segment of the story (Fig. 5b). Using a statistical model (Supplementary Note 1), we analytically explain and calibrate the large increases in SNR that ISFC provides for detecting stimulus-induced correlation patterns. Finally, ISFC revealed reliable and time-varying interactions across brain networks as the story unfolded, indicating that the DMN works in unison with language networks at certain points in the narrative, while exhibiting antagonistic responses at other times (Fig. 6).

The traditional view of DMN function has highlighted its role in intrinsic processes, such as mind wandering, stimulus-independent thought and self-referential mentation^37^,^41^,^42^. More recent studies have begun to implicate the DMN in cognitive functions such as prospective and episodic memory, decision making and social reasoning, and self-generated thoughts^4^,^14^,^15^,^43^,^44^,^45^,^46^,^47^,^48^. Here we demonstrate that, after the removal of intrinsic dynamics, changes in the DMN's correlation patterns over time can be reliably locked to the processing of a narrative as it unfolds (Fig. 4). The locking of DMN states to the narrative was observed both when the DMN was defined at rest using standard seed-based analysis (Figs 3 and 4), and when using *k*-means clustering on the voxel-wise FC correlation matrices (DMN_A_ and DMN_B_, Supplementary Fig. 9)^15^.

Interestingly, the locking of DMN_A_ to the narrative was also observed in the correlation across networks, in particular between the DMN_A_ and the vLANG network (Supplementary Fig. 10). The idea that the DMN is an intrinsic (or task-negative) network predicts that its response dynamics should be anticorrelated with those of other networks that process the extrinsic stimulus. In contrast, the ISFC analysis uncovered a transient switching from negative to positive correlations (and vice versa) between the DMN and language related areas (including low-level auditory areas). Such findings are in agreement with previous electrophysiology work that observed negative correlation between the DMN and attention networks only 20% of the time^40^. Moreover, our results support the idea that the DMN is involved in extrinsic stimulus processing (in this study, an auditory narrative), working cooperatively with other brain regions, in agreement with studies that have implicated the DMN in active semantic processing^49^,^50^.

A central goal of contemporary neuroscience is to determine how the brain dynamically coordinates and integrates information flow across distant brain regions^8^,^51^. Using the ISFC approach, we uncovered robust changes in the correlation structure of the DMN over time that were highly replicable. Within a single brain, the FC patterns during rest and task are non-stationary when measured over a short temporal window, suggesting that brain network states are constantly in flux^6^,^52^. However, since non-stationarity is also observed under anesthesia^7^, it has been unclear whether such fluctuations are due to noise (scanner or physiological noise), or whether they reflect functionally relevant reconfigurations of the neural networks over time^51^. Importantly, although we did observe variability in FC patterns over time within individual subjects during the processing of the story, most of this temporal variation was different in each subject, so that the average of within-subject FC patterns showed little change over time (Supplementary Fig. 7b–d). In contrast, the ISFC analysis revealed a series of stimulus-induced state transitions in the DMN that were replicable across two independent groups of subjects (Fig. 4b, and Supplementary Fig. 7e). The reliable changes in the ISFC patterns of the DMN were not seen during the word scramble condition, suggesting that these changes are related to higher-level processing of the narrative, rather than momentary features of the stimulus, such as individual words. Finally, even at moments in the story when ISFC correlations within the DMN were sparse, the specific configuration was nonetheless highly replicable across independent groups of subjects (Fig. 4, intervals 1 and 3), suggesting that the sparse configuration was meaningfully related to the stimulus. Together these results reveal, for the first time, dynamical changes in the DMN's correlation configuration that are reliable and locked to the processing of a real-life narrative as it unfolds over time.

Our results suggest that the DMN accumulates and integrates information over minutes. Stimulus-induced correlation patterns in the DMN were largest for the intact story, greatly decreased in the paragraph scramble condition, and were almost entirely abolished in the word scramble condition. This implies that the processing timescale of the DMN is longer than the length of single paragraphs (∼40 s) and that information accumulated from prior paragraphs can affect ongoing processing in this system^1^,^2^,^3^,^53^. Furthermore, many studies implicate the DMN in encoding and retrieval of episodic memories^36^,^54^, as well as in high-level cognitive processes such as self-representation^16^,^55^,^56^, prospective planning^16^ and social reasoning^56^. Our study links, for the first time, ongoing changes in the strength of stimulus-induced correlation patterns among the DMN's nodes with fluctuations in the quality of encoding different narrative sections into long-term memory (Fig. 5b). Additional studies are needed to understand the underlying neural mechanisms by which increased connectivity in the DMN is associated with enhanced memory. Possible mechanisms may include global attentional gain, which could increase connectivity across all nodes of the DMN or selectively enhanced correlation between the hippocampus and the DMN^57^.

Isolating stimulus-induced correlations using ISFC echoes the classical electrophysiological distinction between ‘signal correlations' and ‘noise correlations.' Electrophysiologists characterize the functional architecture of a neuronal network by looking at both signal correlations (covariance of mean responses to a signal, which captures shared functional tuning during stimulus processing) and noise correlations (correlation in trial-to-trial variability, which captures effects of attention, learning and memory)^58^. Recent functional magnetic resonance imaging (fMRI) studies have proposed methods for measuring ‘background connectivity' (the neuroimaging version of a noise correlation) by projecting out the mean stimulus-induced responses and calculating within-brain background connectivity^59^,^60^,^61^. ISFC provides a complementary method for measuring signal correlations in BOLD responses to complex time-varying stimuli. Just as for the microcircuits studied in electrophysiology, isolating signal correlations in fMRI is a critical component in characterizing the functional properties of the networks at the macroscopic level (see^62^,^63^ for related considerations). Recent studies have successfully classified cognitive processes based on differences in FC patterns across subjects, across tasks^64^ and across clinical groups^65^,^66^; the present results demonstrate that an even greater and more reliable separation between conditions is achievable with the robust improvement in SNR obtained via ISFC (Figs 3 and 4, Supplementary Figs 3 and 10). Future studies can assess the potential value of ISFC as a diagnostic tool in clinical settings^67^. Furthermore, although ISFC is calculated across brains, it allowed us to isolate stimulus-locked correlation patterns at the single subject level (Fig. 4d).

We have shown that ISFC can uncover positive as well as negative stimulus-induced correlation patterns across regions in the DMN and between networks that are shared across subjects. It is important to note that ISFC cannot detect idiosyncratic stimulus-locked responses that vary across subjects. A possible way to overcome this limitation would be to compute intra-subject functional correlation (within a subject across presentations of identical stimuli)^12^. In addition, further work is needed to understand which features of the stimulus drive the reliable changes in DMN configurations that we observed over the course of a real-life narrative.

To conclude, separating task-induced correlations from intrinsic neural correlations is challenging, but is also crucial for understanding the role of the DMN in information processing. We introduced an ISFC approach to separate stimulus-induced inter-regional correlations from those that arise from ongoing (intrinsic) neural dynamics or physiological noise. Using this technique, we demonstrated for the first time that ISFC dynamics of the DMN were locked to the processing of a real-life narrative as it unfolded over time, and that inter-regional correlations were reliably modulated during active processing of the narrative stimulus. Thus, ISFC provides a reliable method for identifying stimulus-induced dynamics within and between networks, illuminating the role of large-scale brain networks in active information processing.

## Methods

### Experimental procedures

*Subjects*. For the auditory narrative stimulus (‘Pieman'), we scanned 36 subjects who listened to the intact story, 18 of which were used in the primary group (10 females, ages: 18–33) and 18 for the replication group (15 females, ages: 18–31). In the paragraph scramble condition there were 18 subjects (6 males, ages: 18–31). In the word scramble condition there were 36 subjects (20 females, ages: 18–33). In the rest condition there were 36 subjects (15 females, ages: 18–30). For the audio-visual movie (‘Twilight Zone'), there were 24 subjects (13 females, ages: 19–31), and for the block-design paradigm of theory-of-mind group there were 36 subjects (15 females, ages: 21.1±3.5). Another nine subjects were scanned as a control breathing group during both the intact story and rest conditions (breathing group). Additionally, 30 subjects (15 males, ages: 19–31) were recruited for a ‘fill-in-the-blank' memory test conducted outside of the scanner. Procedures were approved by the Princeton University Committee on Activities Involving Human Subjects, and by Western Institutional Review Board (Puyallup, WA). All subjects were native English speakers with normal hearing and provided written informed consent.

*Magnetic resonance imaging (MRI) acquisition*. Subjects were scanned in a 3-T full-body MRI scanner (Skyra; Siemens) with a 16-channel head coil. For functional scans, images were acquired using a T2* weighted echo planer imaging pulse sequence (TR, 1500, ms; echo time, 28 ms; flip angle, 64°), each volume comprising 27 slices of 4 mm thickness; slice-acquisition order was interleaved. In-plane resolution was 3 × 3 mm^2^ (field of view, 192 × 192 mm^2^). Anatomical images were acquired using a T1-weighted magnetization-prepared rapid-acquisition gradient echo pulse sequence (TR, 2300, ms; echo time, 3.08 ms; flip angle 9°; 0.89 mm^3^ resolution; field of view, 256 mm^2^). To minimize head movement, subjects' heads were stabilized with foam padding. Stimuli were presented using the Psychophysics toolbox. Subjects were provided with MRI compatible in-ear mono earbuds (Sensimetrics Model S14), which provided the same audio input to each ear. MRI-safe passive noise-cancelling headphones were placed over the earbuds, for noise removal and safety.

*Collection of physiological signal during the fMRI scans*. Breathing and pulse rate were measured in the intact story and resting state groups, using a belt that was fastened around the chest, and a pulse-oximeter that was attached to the finger. The sampling rate of the breathing signal was 400 Hz, and the pulse meter was sampled at 25 Hz. The signal was transmitted from the scanner bed using a Siemens wireless device (Bluetooth ECG and respiratory sensor, PERU).

*Stimuli and experimental design*. The primary stimuli for the experiment were generated from a 7 min real-life story (‘Pie Man', Jim O'Grady) recorded at a live storytelling performance (‘The Moth' storytelling event, New York City; same stimuli, but not the same subjects, as shown in ref. 2). Subjects listened to the story from beginning to end (intact condition). In addition, subjects listened to scrambled versions of the story, which were generated by dividing the original stimulus into segments of different timescales (paragraphs and words) and then permuting the order of these segments. To generate the scrambled stimuli, the story was segmented manually by identifying the end points of each word and paragraph. Two adjacent short words were assigned to a single segment in cases where we could not separate them. Following segmentation, the intact story was scrambled at two timescales: short—‘words' (W; 608 words, 0.7±0.5 s each) and long—‘paragraphs' (P; 11 paragraphs, 38.1±17.6 s each). Laughter and applause were classified as single word events (4.4% of the words). Twelve seconds of neutral music and 3 s of silence preceded, and 15 s of silence followed, each playback in all conditions. These music and silence periods were discarded from all analyses.

In addition, we used a data set of subjects viewing an audio-visual movie: an episode of ‘The Twilight Zone' entitled ‘The Lateness of the Hour,' 1960 (black-and-white, 25 min long). Subjects (*N*=24) watched the entire movie continuously in a single scan. This data set was collected using the same MRI scanner and protocol as described above. For more details see ref. 57.

Finally, we collected data using a theory-of-mind (Tom) block-design localizer^68^, 4 min 45 s in length. Stimuli consisted of ten stories in each of two conditions: Stories describing false beliefs (Belief) and stories describing photographs and maps (Photo). This data set (*N*=36) was collected using the same MRI scanner and protocol as described above.

*Behavioural test*. The full intact ‘Pie Man' auditory narrative was presented to each subject. After listening, subjects were presented with a printed transcript of the story, with some words or phrases omitted (40 blanks). Subjects were instructed to read the transcript and fill in, as precisely as possible, each of the blanks as they were encountered. The subjects were encouraged to guess if they were unsure of the correct answer. When subjects were unable to venture a guess, they were permitted to not answer and continue with the task. The task ended when subjects reached the end of the transcript. For each subject, the responses to each blank were rated on a scale from 0 to 4. Two raters scored the data and the final rating for each blank was the average across the two raters. A rating of 4 was given when the subject's response exactly matched the omitted words/phrases. A rating of 3 was given when the subject's response was nearly correct, where the idea and words slightly deviated from the perfect response. A rating of 2 was given when the response resembled a general recollection of the correct answer. A rating of 1 was given when the response was completely incorrect. Zero was given when the subject failed to provide a response.

### Data analysis

*Preprocessing*. Functional data were preprocessed and analysed using FSL (www.fmrib.ox.ac.uk/fsl), including correction for head motion and slice-acquisition time, spatial smoothing (6 mm FWHM Gaussian kernel), and high-pass temporal filtering (140 s period). Preprocessed data were aligned to a standard anatomical (MNI152) brain, and interpolated to 3-mm isotropic voxels.

*ROI analysis*. PCC seed-based correlation: we used the posterior cingulate ROI as a seed for defining the DMN in Fig. 2. The ROI was defined functionally and was taken from the literature (PCC; 4-mm radius; MNI coordinates *x*=0, *y*=−53, *z*=26)^28^. We mapped the DMN network by calculating the FC between the PCC seed and the rest of the brain during the rest condition in each of 36 subjects and then averaged across all subjects (Fig. 2a). From the seed-based FC map, we extracted 10 DMN network ROIs. The list of ROIs can be found in Supplementary Table 1.

Clustering-based ROIs: *k*-means clustering was used to extract five networks in each group of 18 subjects, using the FC method (see *k*-means clustering below). Each cluster contained locally connected voxels that were grouped as an ROI. For larger clusters we used a local clustering method to break them into smaller ROIs. Altogether, we defined 52 ROIs across five networks for each group of 18 subjects. The list of ROIs can be found in Supplementary Table 2.

*Surface display*. Projections onto a cortical surface for visualization were performed with NeuroElf (http://neuroelf.net).

*Voxel clustering via neighbourhood correlations*. In order to decompose the large ROIs from the *k*-means clustering into smaller regions, we used the Declustter toolbox (available at http://www.princeton.edu/~fpereira). This algorithm works by computing the maximum of similarities between every voxel and each of its neighbours, and merges the voxel pair with the highest similarity. It replaces the time series for the voxels in that pair with their average, re-computes the correlation with all voxels adjacent to them, and repeats for every voxel.

*Low-frequency respiratory variation and HR variation*. The low-frequency respiratory signal known as ‘respiratory variation' (RV)^33^ was calculated as the s.d. of the respiratory signal over a sliding window of 3 TRs (9 s), where the center of the window is in the middle of the interval. We convolved the RV with the respiratory response function^69^ in a subject-specific manner, for each of the nine subjects that listened to the intact story and participated in a resting condition. HR can also account for BOLD variance^31^. We measured the HR by averaging the inter-pulse intervals over 3 TR intervals, converting it to beats per-minute, and convolving it with the cardiac response function^30^(see Supplementary Fig. 1 for details).

*Head motion trajectory*. We calculated the instantaneous head motion for each subject in the breathing group (*n*=9) as the sum of the absolute displacement (derivative) in the six motion parameters, which were estimated during the motion correction step (translations: Δ*x*, Δ*y*, Δ*z*; rotations:α, β, γ). The rotational displacements were converted from degrees to millimeters^35^.

*Removal of non-neuronal signal sources*. Slow changes of respiration over time (RV) have been shown to induce robust changes in the BOLD signal^33^ in many areas around the cerebral midline, including parts of the DMN. Therefore, we used multiple linear regression to project out three nuisance variables from the BOLD data. This was performed separately for each subject and for all conditions before calculating FC or ISFC. Nuisance regressors were: (1) the average time course of high s.d. voxels outside the grey matter mask (voxels in the top 1% largest s.d., likely blood vessels^70^ known to be correlated with RV); (2) the average BOLD signal measured in cerebrospinal fluid; (3) the average white matter signal. All masks (grey matter, white matter and cerebrospinal fluid) were obtained from the probabilistic FSL atlas in standard MNI space (>95% region probability, Harvard–Oxford cortical and subcortical structural atlases).

*Statistical analysis of time-series correlations*. Because of the presence of long-range temporal autocorrelation in the BOLD signal, the statistical likelihood of each observed correlation was assessed using a permutation procedure based on surrogate data (bootstrapping procedure). The surrogate data was generated using phase randomization. Phase-randomized surrogates have the same mean and autocorrelation as the original signal. For the ISFC analysis, the null hypothesis was that the BOLD signal in each voxel in each individual was independent of the BOLD signal values in all other voxels in any other individual at any point in time. For the FC analysis. the null hypothesis was that the BOLD signal in each voxel in each individual was independent of the BOLD signal values in all other voxels within the same individual at any point in time. For all conditions, we applied Fast Fourier Transform to the signal. To randomize the phases, we multiplied each complex amplitude by *e*^*jϕ*^, where *ϕ* is independently chosen for each frequency from the interval (0, 2*π*). In order for the inverse Fourier transform to be real (no imaginary components), we symmetrized the phases, so that *ϕ*(*f*)=−*ϕ*(−*f*). Finally, the inverse Fourier transform is the surrogate data.

For each surrogate data set, all metrics (seed-based ISFC/FC, network-based ISFC/FC and sliding window ISFC/FC) were applied to the surrogate data in the same manner as for the empirical data. Using Monte Carlo simulations, we generated null distributions of correlations both across subjects (ISFC) and within subjects (FC).

To correct for multiple comparisons, we selected the maximum ISFC or FC value from the null distribution of all voxels in a given iteration of the permutation procedure. We repeated this procedure 10,000 times to obtain a null distribution of the maximum noise correlation values. We controlled the FWER by defining a threshold (R*) at the q*100th percentile of the null distribution of maximum values. In other words, in the FC and ISFC maps (Fig. 2), only voxels with correlation value (*R*) above the threshold derived from the bootstrapping procedure (R*) were considered significant after correction for multiple comparisons and were presented on the final map. The thresholds for each condition were as follows (all for *q*<0.01): FC (*all conditions*) R*=0.25; ISFC (*intact story*) R*=0.13; ISFC (*rest, word scramble*) R*=0.1.

*Network-based ISFC*. We defined the ISFC correlation matrix between all nodes (voxels or ROIs) across brains in the following manner. The neural signals *X*_*i*_ measured from subject *i*, *i*=1,...,*k*, are in the form of a *p* × *n* matrix that contains signals from *p* neural sources over *n* time points. All time courses were *z*-scored within subjects to zero mean and unit variance. Thus, the subject-based ISFC was calculated by the Pearson correlation between single subject and the average of all other subjects as:


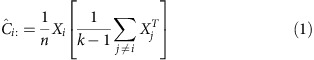


Hence, the *p* × *p* group-based ISFC matrix was given by:


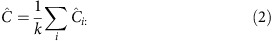


Fisher's *r*-to-*z* transformation was applied to each correlation coefficient before averaging, in order to increase normality of the distribution of correlation values, and averaged *z* values were then inverse transformed (*z*-to-*r*) to produce average *r*-values. The final ISFC matrix is given by 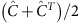. We imposed this symmetry since we consider the correlation between two brain regions as unidirectional, as in FC. The diagonal of this group-based ISFC matrix is the inter-subject correlation^26^, which is the correlation between the same ROIs across subjects.

*Seed-based ISFC*. Seed-based ISFC is a special case of network-based ISFC: it is the Pearson correlation between the response time course in one brain region in one subject and the average response time courses in all voxels of all other subjects. Hence, seed-based ISFC is a one-dimensional correlation map of length *p* (that is, it is a single row of the *p* × *p* ISFC matrix).

*Network-based and seed-based FC*. Network-based and seed-based FC were calculated in the same manner as network-based and seed-based ISFC, except that the correlations were calculated within each subject and then averaged across all subjects in a group.

*Network correlation patterns over time*. Network-based ISFC was calculated over a sliding window *t*_win_, 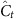, within each time interval (*t,t+t*_win_). We defined the network correlation pattern of a group, at time *t*, as the lower off-diagonal terms of the symmetric correlation matrix. The mean network correlation, at time interval (*t,t+t*_win_) was defined as the mean of the lower off-diagonal terms of the correlation matrix.

*Reliability of network correlation patterns (‘Network States') over time*. For each condition (rest, word scramble, intact story), we randomly partitioned the group of 36 subjects into two independent groups of 18 subjects, and computed the ISFC patterns in the DMN nodes at each sliding window in each group. To assess the reliability of the network's state (‘fingerprints') we correlated the ISFC patterns across the two independent groups at each sliding window. We repeated this procedure 100 times, beginning each time with a new random partition, and calculated the mean correlation and the s.d. of the mean across the 100 iterations (Fig. 4c).

*Network visualization*. For data collected from p regions, the *p*x*p* correlation matrix was represented in a graph with p nodes (each node is an ROI) and *p*(*p*−1)/2 edges (correlations between ROI time courses). The lower-triangular terms of 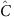 provided the edges of the network. The diagonal of the matrix is the ISC between the same nodes across subjects. Each node was represented by a circle whose diameter scaled with the ISC of that node (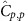). The thickness of lines in the diagram indicates the strength of edges of the network, 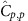 (line width=60 × 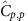). Small negative values (−0.1<*r*<0) were set to zero, using this graphic representation. We used the open source program Cytoscape (http://www.cytoscape.org/) to visualize the networks.

*Behavioural test analysis*. The average memory score across subjects for each ‘blank' (out of forty blanks) was calculated and then smoothed using local regression smoothing with a span of 30 TRs (using Matlab's *smooth* function with the ‘loess' option). The same span was used to calculate the ISFC over a sliding window of 30 TRs. To calculate the significance of the correlation between the average memory score over time and ISFC in the DMN over time, we used the phase randomization procedure (see *Statistical analysis*) for each memory score time course in each subject, and repeated the procedure 1000 times to generate a null distribution.

*Clustering within the FC and ISFC spaces*. We computed the ISC across the 36 subjects who listened to the intact story. In order to identify the most reliable voxels, we extracted the top 15% of ISC values using a threshold of *r*=0.20 (8672 voxels). Voxels that did not respond reliably to the auditory narrative (that is, those with below-threshold ISC) were removed from the analysis. We next divided the 36 subjects into two groups of 18 subjects (group 1 and group 2) and computed the within-group FC or ISFC matrices. The correlation matrix consists of 8672 voxel-based correlation maps across subjects. To assess the structure of ISFC and FC patterns we applied the *k*-means clustering algorithm to the covariance matrix. For each *K* (number of clusters) we performed the clustering separately in group 1 and group 2, and then assessed reproducibility by calculating the spatial overlap of each cluster in group 1 with all other clusters in group 2, using the S*ϕ*rensen-Dice index


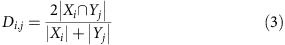


Where, *X*_*i*_ and *Y*_*j*_ are the clusters from group 1 and group 2, respectively. *D* is a *K* × *K* matrix, and our criterion was to choose *K* that maximized the Dice indices for each row of the matrix D.

*k-means clustering*. The *k*-means clustering was performed using the *kmeans* function in MATLAB (Mathworks, 2012). Briefly, the goal of this *k*-means procedure is to partition the voxel-based ISFC and FC correlation matrix into *k* mutually exclusive clusters. Each cluster is defined by a set of N member voxels (each with an associated correlation vector) and by the centroid of the correlation vectors in the cluster. The iterative algorithm minimizes the sum of distances from each voxel (vector) to its cluster centroid, overall clusters. We used the L2 distance (Euclidean distance) function, although very similar results were obtained using the L1 norm (Manhattan distance), which may be superior for high-dimensional data sets.

### Data availability

The data that support the findings of this study are available from the corresponding author upon request.

## Additional information

**How to cite this article:** Simony, E. *et al*. Dynamic reconfiguration of the default mode network during narrative comprehension. *Nat. Commun.* 7:12141 doi: 10.1038/ncomms12141 (2016).

## Supplementary Material

Supplementary Information Supplementary Figures 1-11, Supplementary Tables 1-2, Supplementary Notes 1-2, Supplementary References.

Supplementary Movie 1 Voxel-wise ISFC reveals fine-grained stimulus-dependent interaction within and across networks. The covariance matrix (8672x8672 voxels) was calculated over sliding window intervals of 90 seconds (1TR shift).
