# Supplementary material for: Dynamic reconfiguration of the default mode network during narrative comprehension
Source: Nat Commun. 2016 Jul 18;7:12141. doi: 10.1038/ncomms12141 (PMC4960303; doi:10.1038/ncomms12141)
Supplement: Supplementary Information — Supplementary Figures 1-11, Supplementary Tables 1-2, Supplementary Notes 1-2, Supplementary References. [file ncomms12141-s1.pdf]

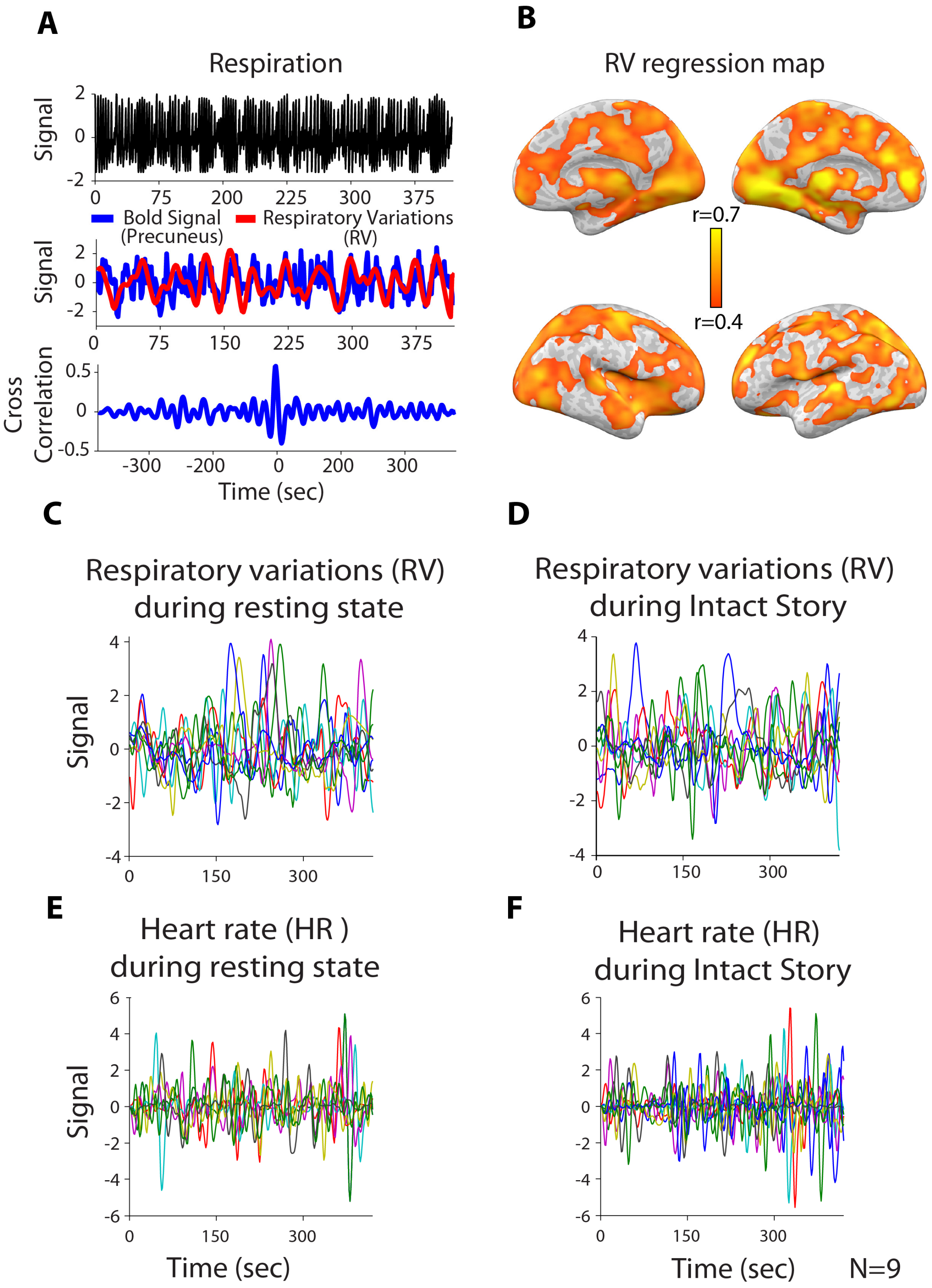
A
Respiration
Signal
Bold Signal (Precuneus)
Respiratory Variations (RV)
Cross Correlation
Time (sec)
B
RV regression map
r=0.7
r=0.4
C
Respiratory variations (RV) during resting state
D
Respiratory variations (RV) during Intact Story
E
Heart rate (HR) during resting state
F
Heart rate (HR) during Intact Story
Time (sec)
N=9

Supplementary Figure 1 | ISFC filters out non-neuronal correlations. During both rest and task conditions, ISFC is unaffected by the three major non-neuronal sources of noise: heart rate [1,2], respiration [1,3] and head motion [4]. Previous studies have shown that heart rate (HR) and slow changes in breathing depth and rate over time, known as the respiratory variation (RV), could account for significant variance in the gray matter signal during rest [5]. Here we measured the breathing and heart rate, and used the RVHRCORR model [1] to extract the low-frequency RV and HR during rest and while 9 subjects listened to the intact story (for details see Methods, "Low frequency respiratory and heart rate variation"). In agreement with previous reports, RV correlated with many brain areas within individuals during rest and during the story condition. **(A)** Cross-correlation (r~=0.5) between the RV and BOLD signal in the precuneus for one individual subject during rest. **(B)** The correlation between the RV and BOLD signal within the brain of an individual subject during rest is high. **(C, D)** The RV signals were uncorrelated across the 9 subjects during both the rest condition (r=-0.004, p>0.2) and the story condition (r=-0.02, p>0.18), validating our assumption that these non-neuronal artifacts can influence the FC analysis but not the ISFC analysis. Interestingly, by regressing out the RV signal from the precuneus timecourse and from early auditory areas (A1), we got a 20% increase in the inter-subject correlation (ISC) in both areas. This suggests that by filtering out physiological noise for each subject during the preprocessing stage, we can better expose the stimulus-induced signal that is shared across subjects and revealed by the ISFC method. **(E,F)** Similarly, we found that the HR signals and head-motion trajectories were not correlated across subjects during rest (r< 0.04, p>0.26) or the story condition (r< 0.02, p>0.3). Together, these results provide empirical evidence for our hypothesis that the intrinsic neuronal signal and non-neuronal noise can induce high FC values within a brain, but no ISFC across subjects. This finding supports the ISFC equation in Figure 1D, where the ISFC correlation approaches zero in cases where I and N dominate the signal and S_1 and $\tilde{S}_2$ are set to zero.

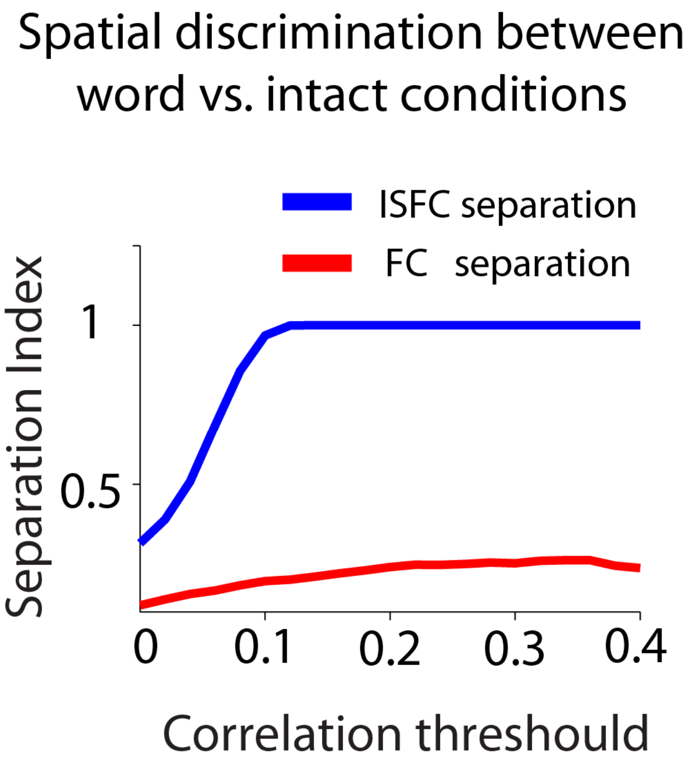

Supplementary Figure 2 | Spatial discrimination between seed-based ISFC maps during word-scramble and intact story conditions.

The separation index (1-Dice index) is a measure of separation between conditions, where 0 indicates no separation, and 1 indicates absolute separation. For a given correlation threshold, we computed the separation index between the word-scramble and intact story maps for both the ISFC and FC methods. Using ISFC, we see a clear separation between conditions for all thresholds above r>0.1.

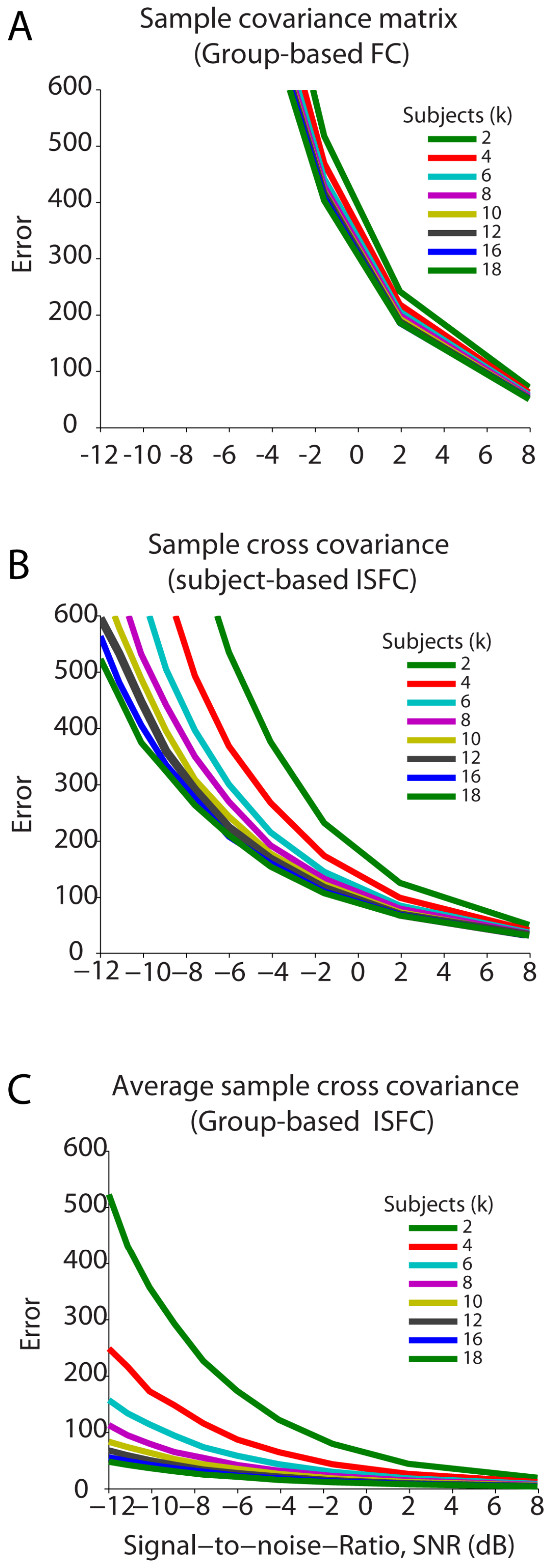

A
Sample covariance matrix
(Group-based FC)
Error
600
500
400
300
200
100
0
-12 -10 -8 -6 -4 -2 0 2 4 6 8
Subjects (k)
2
4
6
8
10
12
16
18
B
Sample cross covariance
(subject-based ISFC)
Error
600
500
400
300
200
100
0
−12 −10 −8 −6 −4 −2 0 2 4 6 8
Subjects (k)
2
4
6
8
10
12
16
18
C
Average sample cross covariance
(Group-based ISFC)
Error
600
500
400
300
200
100
0
−12 −10 −8 −6 −4 −2 0 2 4 6 8
Signal−to−noise−Ratio, SNR (dB)
Subjects (k)
2
4
6
8
10
12
16
18

Supplementary Figure 3 | Gain in SNR for decoding the shared covariance matrix using inter-subject functional correlation (ISFC) and functional connectivity (FC). The simulation is based on our statistical model and supports the analytical results (see supplementary note 1). SNR was calculated in dB, as the ratio between the shared signal variance, $Tr[AA^T]$ and the sum of two variances: (i) idiosyncratic (stimulus-dependent) variance σ_D^2 and (ii) Intrinsic signal variance $Tr[Q]$. **(A)** Group-based functional connectivity (FC) for estimating the joint stimulus-induced covariance matrix, across the group, as a function of SNR and number of subjects. Changing the number of subjects does not substantially affect performance. **(B)** Subject-based ISFC: the ISFC between a single subject and the rest of the group. This measure is used for classification at the level of single subjects (e.g. Fig 4D). **(C)** Average of subject-based ISFC across the group yields the group-based ISFC. This estimate best approximates the true shared stimulus-induced covariance matrix (see supplementary note 1).

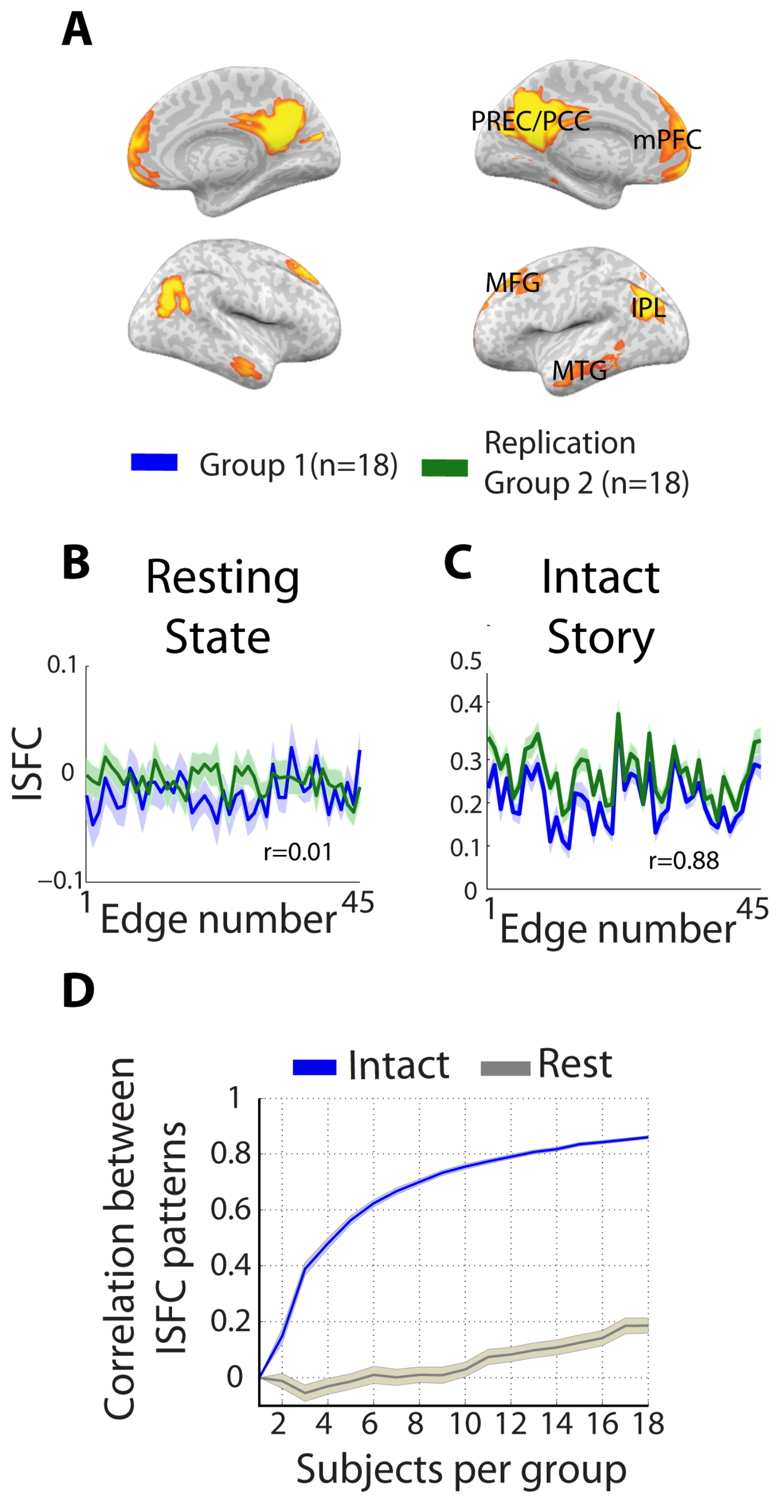

A
PREC/PCC
mPFC
MFG
IPL
MTG
Group 1(n=18)
Replication
Group 2 (n=18)
B
Resting
State
ISFC
0.1
0
−0.1
r=0.01
1
Edge number
45
C
Intact
Story
0.5
0.4
0.3
0.2
0.1
0
r=0.88
1
Edge number
45
D
Intact
Rest
Correlation between
ISFC patterns
1
0.8
0.6
0.4
0.2
0
2
4
6
8
10
12
14
16
18
Subjects per group

Supplementary Figure 4 | Replication of DMN correlation patterns across two independent groups using ISFC. (**A**) The DMN map as defined from the resting state data. (**B, C**) ISFC DMN edge correlations are not similar across two independent groups (blue, green) in the resting-state data (r=0.01), but they become much more similar in the intact story condition (r=0.88). (**D**) The number of subjects needed in order to isolate reliable ISFC patterns for the intact story (blue) and rest (gray) conditions.

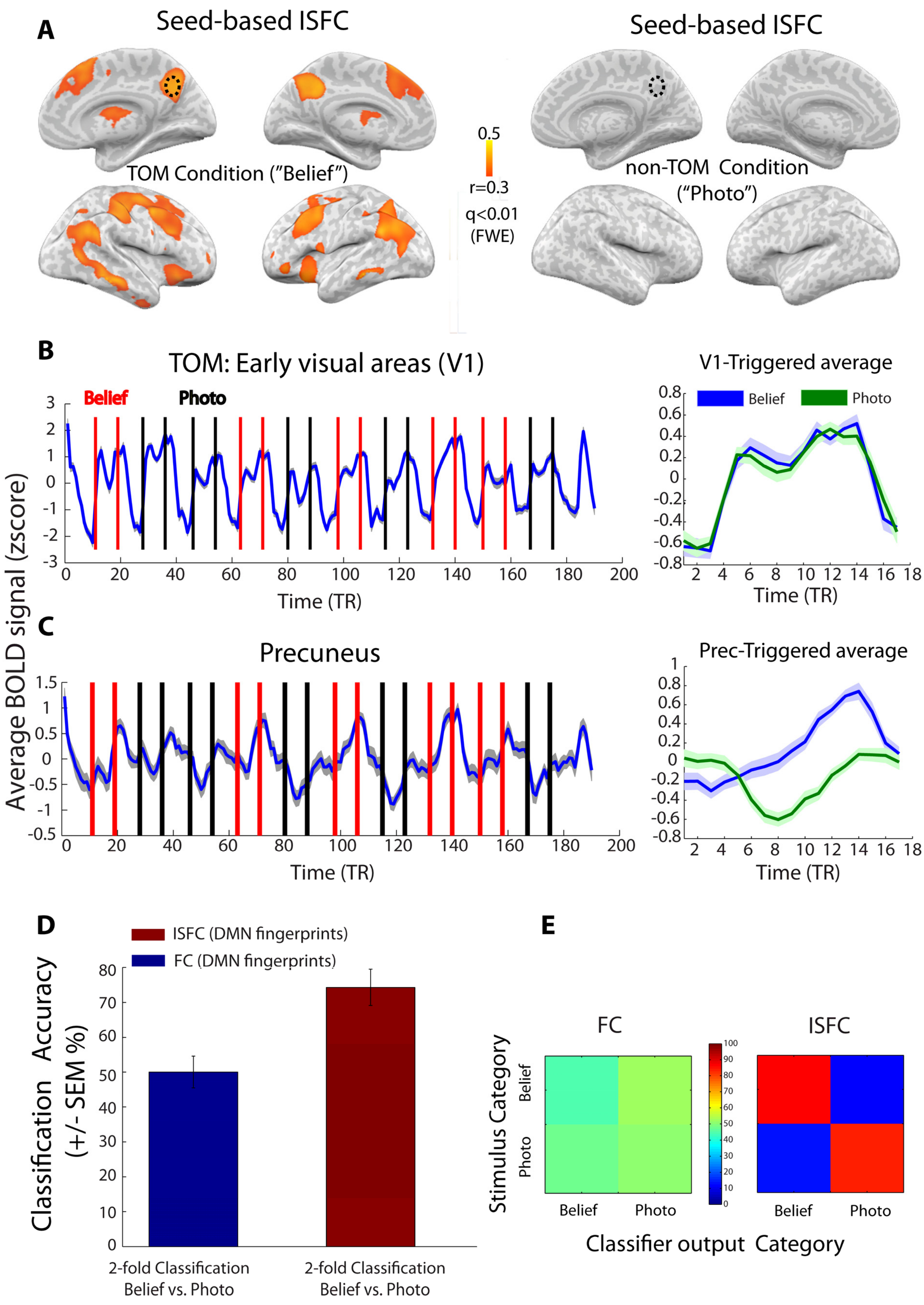
A
Seed-based ISFC
TOM Condition ("Belief")
0.5
r=0.3
q<0.01
(FWE)
Seed-based ISFC
non-TOM Condition
("Photo")
B
TOM: Early visual areas (V1)
Belief
Photo
Time (TR)
V1-Triggered average
Belief
Photo
Time (TR)
Average BOLD signal (zscore)
C
Precuneus
Time (TR)
Prec-Triggered average
Time (TR)
D
ISFC (DMN fingerprints)
FC (DMN fingerprints)
Classification Accuracy
(+/- SEM %)
2-fold Classification
Belief vs. Photo
2-fold Classification
Belief vs. Photo
E
FC
ISFC
Stimulus Category
Belief
Photo
Belief
Photo
Belief
Photo
Classifier output Category

Supplementary Figure 5 | Classification of intervals in a block-design theory-of-mind (TOM) task using DMN fingerprints. To test ISFC classification performance in a block design paradigm we ran a theory of mind (ToM) localizer paradigm on 36 subjects. The ToM is a standard localizer[6], developed to localize high-order areas which respond more strongly when subjects read a paragraph that requires an inference about beliefs held by different protagonists ("Belief" or "ToM" condition, 10 stories, each ~10sec, 5 repetitions) vs. stories describing photographs and maps with no belief content ("Photo" or "non-ToM" condition, 10 blocks, each ~10sec, 5 repetitions). First, using seed-based ISFC (seed in the precuneus), during the belief condition, we localized a set of brain areas including the temporal parietal junction, precuneus and middle medial prefrontal cortex (**A**, left panel). By contrast, during the non-TOM condition we did not observe any reliable ISFC in the DMN (**A**, right panel). Average BOLD activity over 36 subjects in early visual regions (V1), using a standard block-design TOM experiment. Alternating intervals of stories describing false beliefs (Belief, red) and stories describing photographs and maps (Photo, black). Condition blocks were separated by fixation periods. Event-triggered average signal in V1 over the two conditions, showing the same response for both non-TOM and TOM conditions (**B**). There was increased activity in the precuneus during the ToM condition (**C**). Next, we used the exact same DMN nodes, defined using FC at rest for the previous analyses, to classify the conditions within each subject (ToM vs. no-ToM concatenated blocks) based on the ISFC correlation matrix and FC correlation matrix. For each subject, signals from all intervals in a given condition were concatenated, resulting in 2 time courses (one for each condition) per DMN node per subject. Using across-subject classification and a leave-one-out procedure, training on 35 subjects (see above), we found that in this traditional block design experiment, ISFC classification was far superior to FC (**D**; ISFC 74.1±4.2% vs. FC 50±4.5%, chance level 50%).(**E**) Confusion matrices for both FC and ISFC across two conditions.

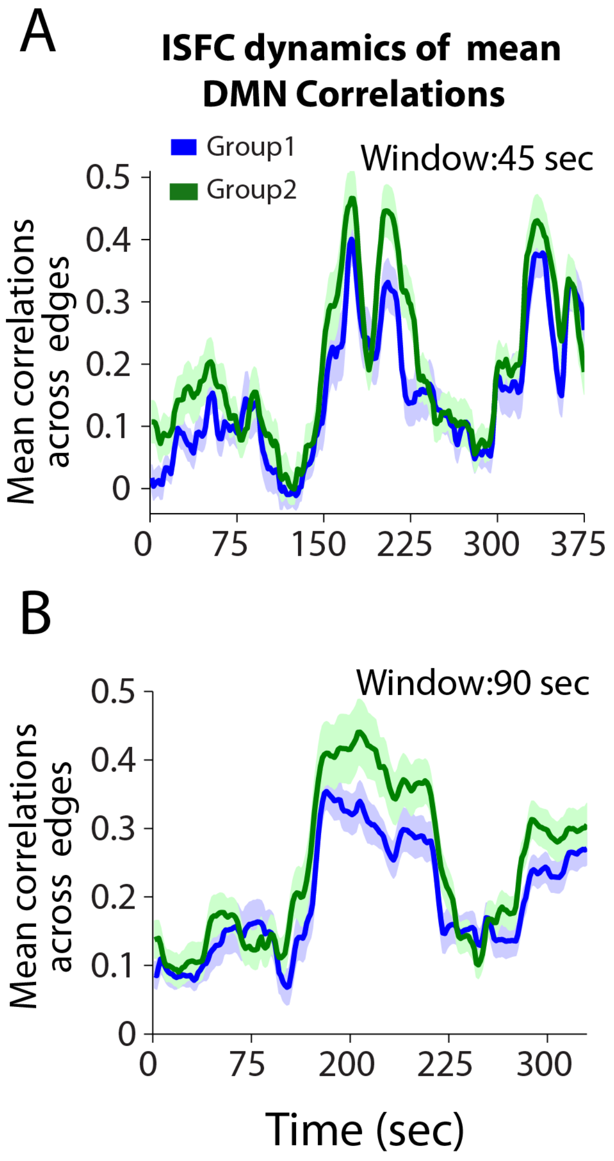

Supplementary Figure 6 | Reliable ISFC dynamics over shorter and longer temporal windows during the intact story.

(A) Reliable dynamics of the mean ISFC within the DMN network across two independent groups of 18 subjects (with DMN member voxels defined using resting state FC). ISFC is computed using a sliding window of 45 s (30 TRs), in steps of 1 TR **(B)** The same as in (A) using a sliding window of 90s (60 TRs).

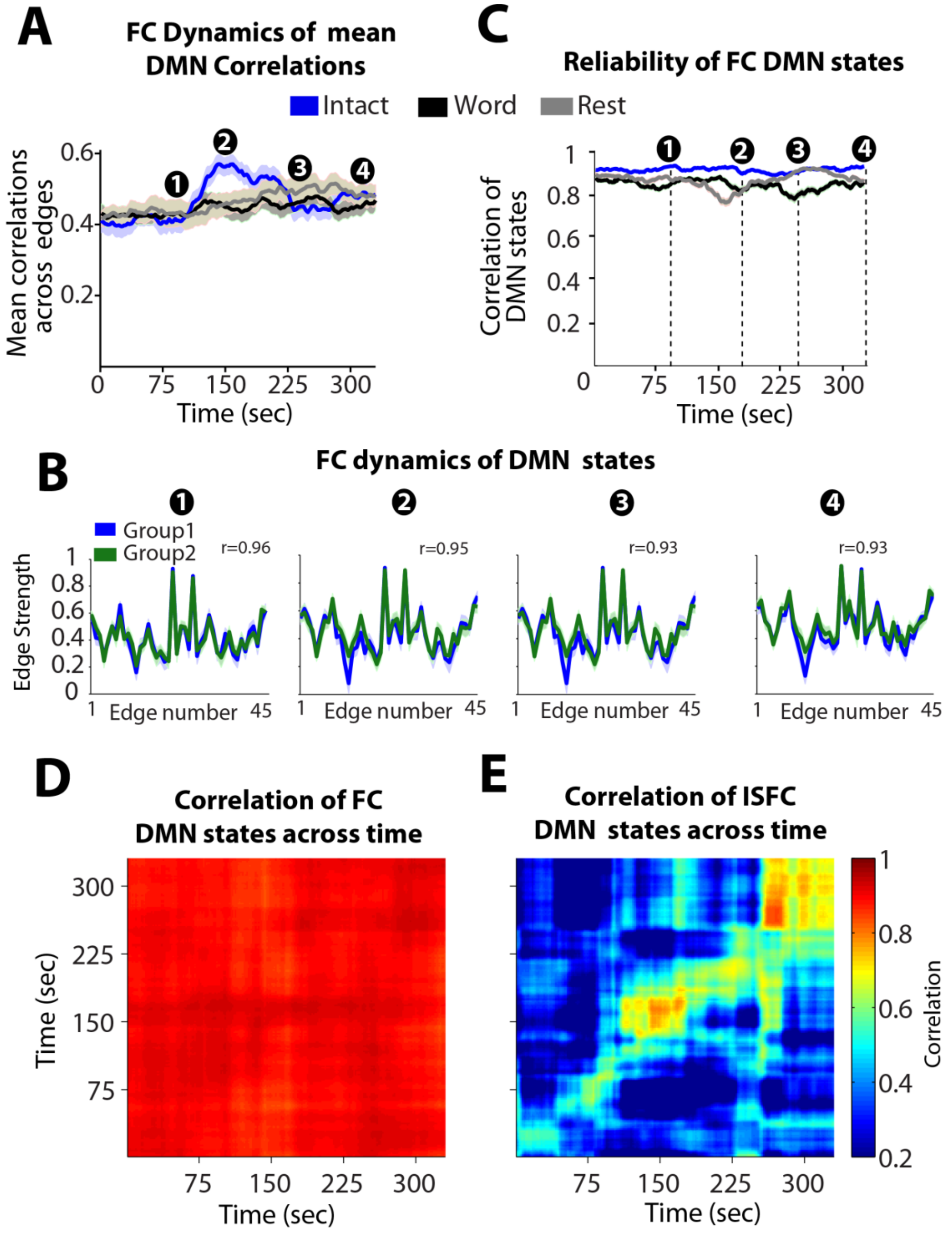
A
FC Dynamics of mean DMN Correlations
Intact
Word
Rest
Mean correlations across edges
0.6
0.4
0.2
0
75
150
225
300
Time (sec)
C
Reliability of FC DMN states
Correlation of DMN states
1
0.8
0.6
0.4
0.2
0
75
150
225
300
Time (sec)
B
FC dynamics of DMN states
Group1
Group2
r=0.96
r=0.95
r=0.93
r=0.93
Edge Strength
1
0.8
0.6
0.4
0.2
0
1
Edge number
45
D
Correlation of FC DMN states across time
Time (sec)
300
225
150
75
75
150
225
300
Time (sec)
E
Correlation of ISFC DMN states across time
75
150
225
300
Time (sec)
Correlation
1
0.8
0.6
0.4
0.2

Supplementary Figure 7 | FC DMN correlation patterns during the intact story are slightly modulated by the stimuli.

(A) Mean FC DMN edge correlations over time (90 s sliding window) during the intact story (blue), word-scramble (black) and rest (gray) conditions, averaged over eighteen subjects. **(B)** DMN correlation patterns (states) over four intervals (1-4) during the intact story condition in group 1 (eighteen subjects, blue) and in replication group 2 (eighteen subjects, green) **(C)** High reliability of standard FC correlation patterns in the DMN, computed over time during the Intact Story, Word Scramble and resting state conditions. **(D)** Correlation of the FC patterns across different time windows indicates that they are very similar over the course of the story. **(E)** Correlation of the ISFC patterns across different time windows indicates that the patterns differ more systematically as the narrative unfolds.

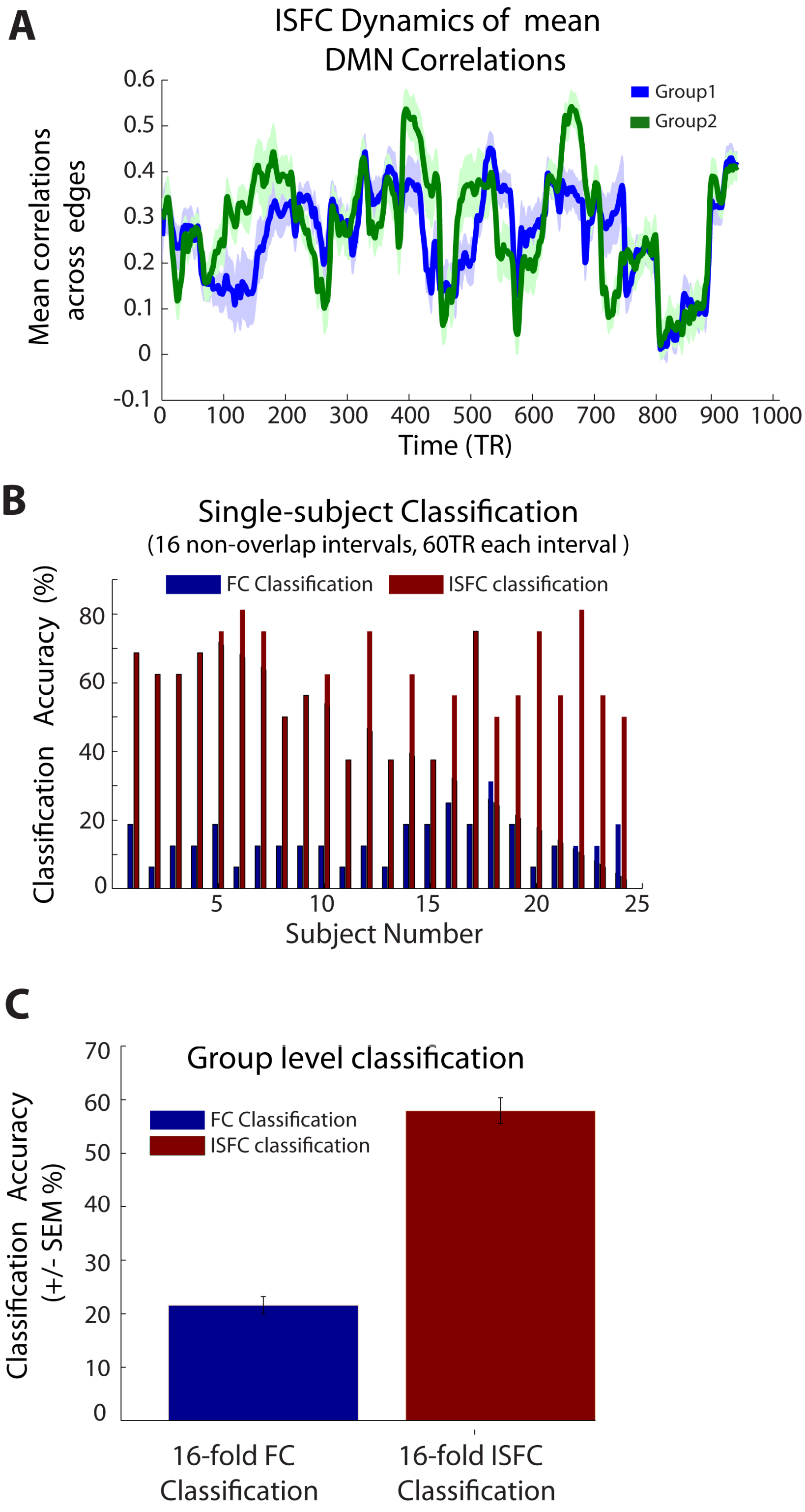
A
ISFC Dynamics of mean DMN Correlations
Mean correlations across edges
Time (TR)
Group1
Group2
0.6
0.5
0.4
0.3
0.2
0.1
0
-0.1
0
100
200
300
400
500
600
700
800
900
1000
B
Single-subject Classification
(16 non-overlap intervals, 60TR each interval)
FC Classification
ISFC classification
Classification Accuracy (%)
Subject Number
0
20
40
60
80
5
10
15
20
25
C
Group level classification
FC Classification
ISFC classification
Classification Accuracy (+/- SEM %)
0
10
20
30
40
50
60
70
16-fold FC Classification
16-fold ISFC Classification

Supplementary Fig 8 | Replicability of DMN ISFC dynamics during a 25-minute audiovisual movie, and classification of DMN fingerprints. To what extent were the modulations in DMN correlation over time dependent on the specific narrative stimulus that we used? To test this we calculated ISFC within the DMN for an additional dataset of 24 subjects who watched a 25-minute audiovisual television episode (from "The Twilight Zone"; for more information see 7). We split the data into 2 groups of 12 subjects, and calculated the mean ISFC over time (60 TRs sliding-windows) in the DMN within each group. Similar to the auditory-only story ("Pieman"), we observed reliable modulations of the mean ISFC pattern over time for the audio-visual movie ($r=0.62, p<10-6$). However, the changes in the mean ISFC over time during the audio-visual movie had a unique temporal trajectory, distinct from the temporal trajectory observed for the auditory-only stimulus (correlation of time-course of mean ISFC across different stimuli: $r<0.07, p<0.26$, maximum df = 220). **(A)** Mean ISFC of all edges in the DMN computed over time in 90-second sliding windows (window at time t is data from [t, t+90s] with a step-size of 1.5 s between windows). The ISFC across 12 subjects is shown for the intact movie (blue), and for the replication group of 12 subjects (green). **(B)** Across-subject classification of DMN fingerprints, using ISFC and FC, over 16 non-overlapping intervals (16 x 90sec) at the level of single subjects. **(C)** Mean ISFC and FC interval classification across 24 subjects (chance level = 6.25%)**.**

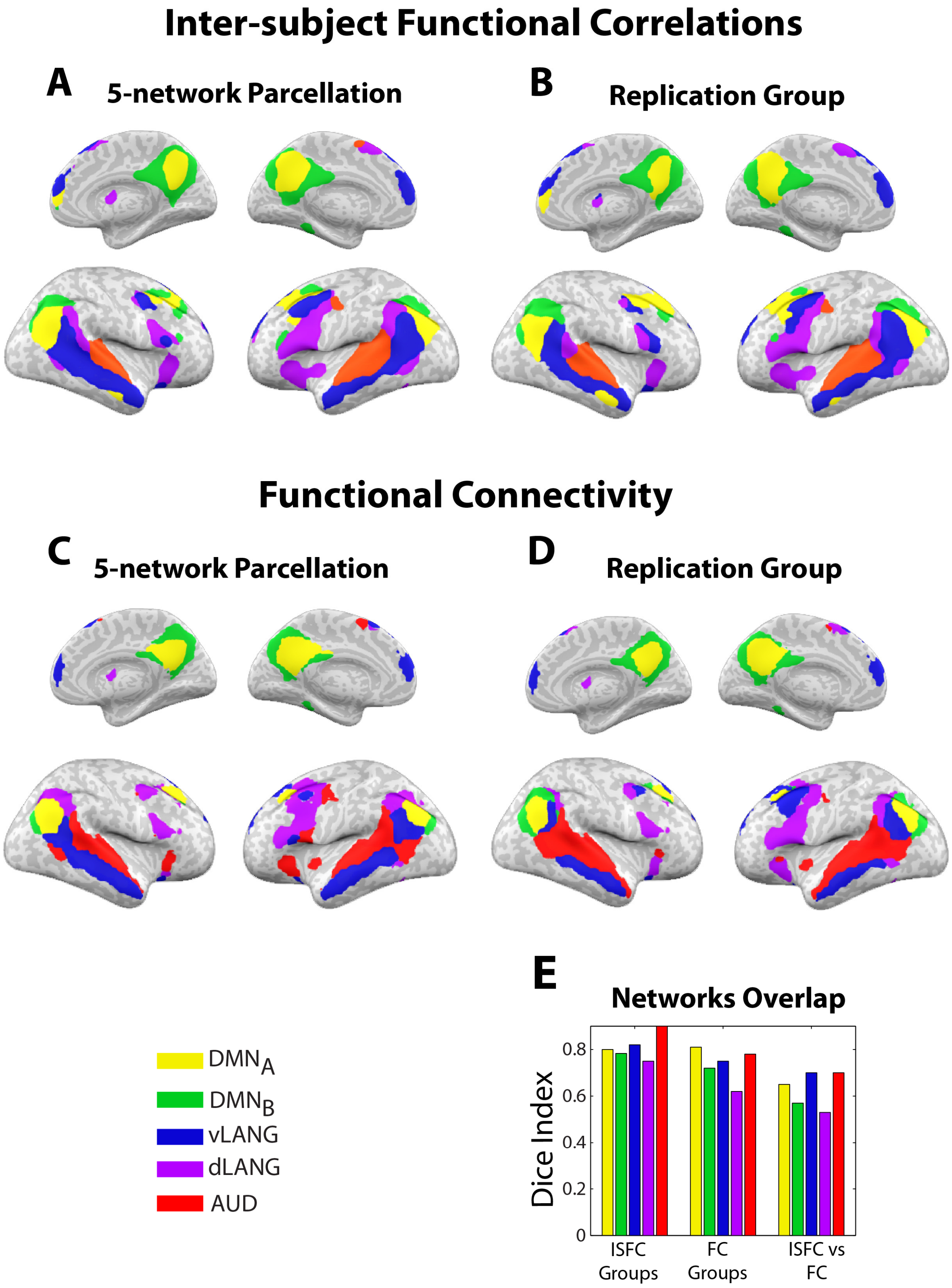
Inter-subject Functional Correlations
A
5-network Parcellation
B
Replication Group
Functional Connectivity
C
5-network Parcellation
D
Replication Group
E
Networks Overlap
Dice Index
0
0.2
0.4
0.6
0.8
ISFC Groups
FC Groups
ISFC vs FC
DMNA
DMNB
vLANG
dLANG
AUD

Supplementary Figure 9 | Cluster analysis of the ISFC covariance matrix during the intact story and the FC covariance matrix during rest reveals similar network organization structure.

(A,B) K-means clustering of the voxel-based ISFC covariance matrix during the intact-story (K=5) reveals five networks that are reliably extracted across two independent groups of eighteen subjects. The cluster surrounding the Heschl's gyrus was labeled the auditory network (AUD, red). The DMN seems to be split into two sub-networks labeled DMN_A (yellow) and DMN_B (green). This division is compatible with prior publications that divide the DMN into two sub-networks: the core network and the medial temporal lobe (MTL) subsystem[8,9]. Finally, the two other networks resembled the division between the ventral and dorsal language streams proposed by Hickok and Poeppel[10,11] and were labeled, provisionally and tentatively, as ventral language network (vLANG , blue) and dorsal language network, respectively (dLANG, purple). **(C,D)** k-Means clustering (k=5) of the voxel-based FC covariance matrix during resting-state reveals similar networks across two independent groups of eighteen subjects. **(E)** The Dice index indicates the similarity of the networks identified within and across the FC and ISFC methods and across the original and replication groups.

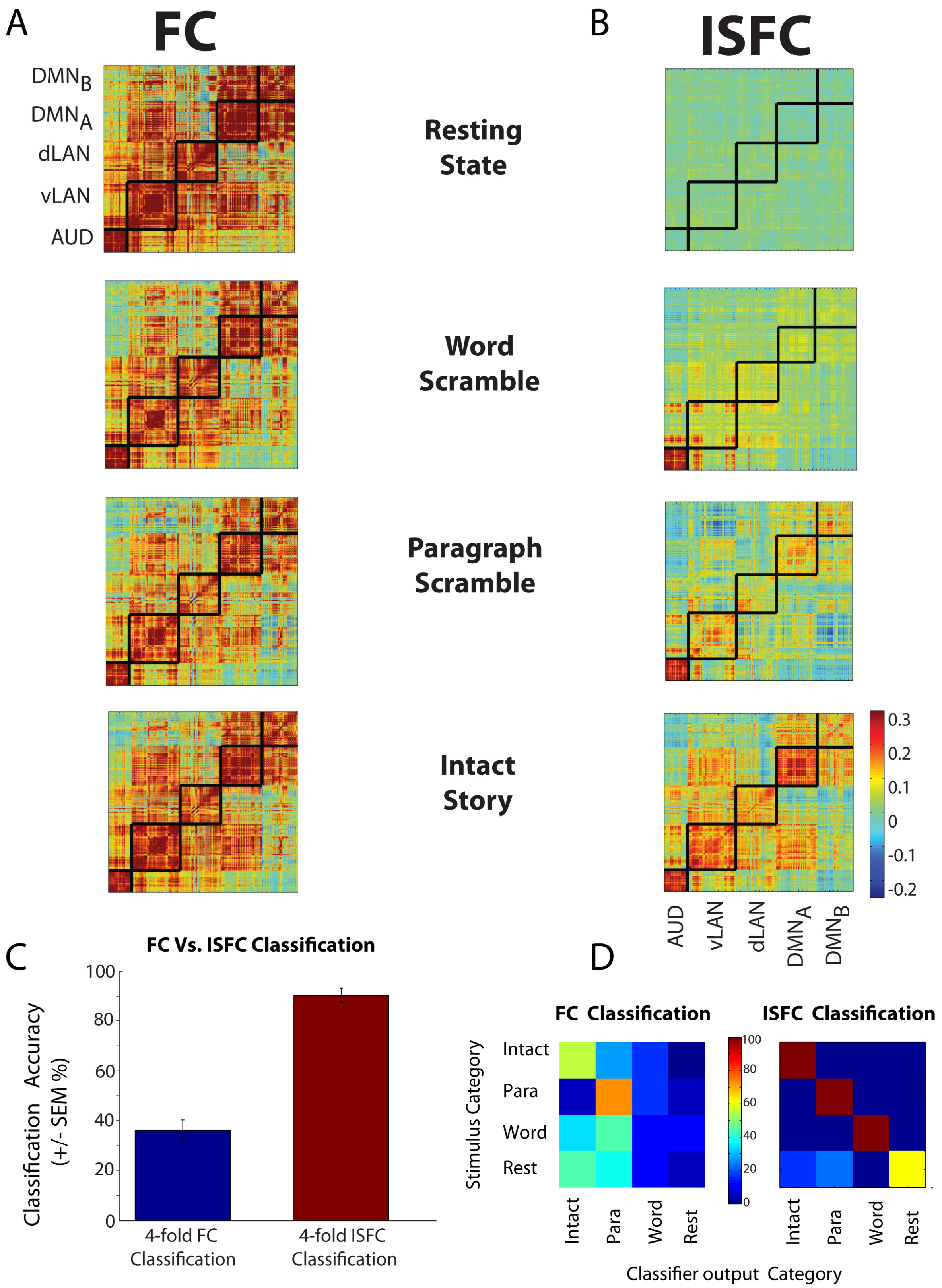
A
FC
B
ISFC
DMNB
DMNA
dLAN
vLAN
AUD
Resting State
Word Scramble
Paragraph Scramble
Intact Story
0.3
0.2
0.1
0
-0.1
-0.2
AUD
vLAN
dLAN
DMNA
DMNB
C
FC Vs. ISFC Classification
Classification Accuracy (+/- SEM %)
100
80
60
40
20
0
4-fold FC Classification
4-fold ISFC Classification
D
FC Classification
ISFC Classification
Stimulus Category
Intact
Para
Word
Rest
100
80
60
40
20
0
Intact
Para
Word
Rest
Classifier output Category

Supplementary Figure 10 | Voxel-Based ISFC correlation matrices reveal fine-grained stimulus-dependent interaction within networks. We tested whether ISFC analysis can be extended to measure the stimulus-induced inter-regional correlation patterns between the DMN and other functional networks. Here we present the FC and ISFC correlation matrices for all voxels (8672x8672 voxels, 18 subjects) that responded reliably to the intact narrative (i.e., significant ISC along the diagonal of the matrix, see Methods). The correlation matrix was re-ordered by the output of a K-means clustering algorithm[12] performed on the FC matrix during rest. The algorithm found five consistent FC networks that could be replicated across two groups (Supplementary Fig. 9B). The cluster surrounding Heschl's gyrus was labeled the "auditory network" (AUD, red). The DMN appeared to be split into two sub-networks, labeled DMN_A (yellow), and DMN_B (green). This division is compatible with prior publications that divide the DMN into two sub-networks: the core network and the medial temporal lobe (MTL) subsystem[8,9]. **.**Finally, because the remaining two networks resembled the division between the ventral and dorsal language streams proposed by Hickok and Poeppel[10,11], they were labeled, provisionally and tentatively, as ventral language network (vLANG , blue) and dorsal language network, respectively (dLANG, purple). Stimulus-induced correlation was observed between networks, and the inter-network correlation was especially pronounced when subjects listened to the intact story. Correlation matrices were calculated in the four conditions: Resting State, Word Scramble, Paragraph Scramble, and Intact Story conditions. The average FC correlation matrices across all voxels were quite similar across all 4 conditions **(A)**. In contrast, the voxelwise ISFC correlation matrix showed clear changes across stimuli **(B)**. The ISFC correlation matrix was empty during rest, but during the Word Scramble condition we observed reliable ISFC in the auditory network and subsets of the language networks. In the Paragraph Scramble condition, there was an increase in ISFC within the language networks and in the DMN network. Finally, the stimulus-induced ISFC was maximized within all networks during the processing of the intact story (see Supplemental Movie 1 for voxelwise network dynamics over time within the intact story). In order to quantify the separation between different conditions in each network, we plot the normalized edge strength histograms of ISFC and FC for each network across four conditions (Supplementary Fig. 11). In addition, we computed the four-fold across-subject classification between the ROI-based correlation matrices (52x52) (Supplementary Table 2), during four conditions: Intact Story, Paragraph Scramble, Word Scramble, and Resting State, using FC and ISFC. ISFC showed a 54% increase in classification accuracy over FC (90±2.9% vs. 36±4.1%); **(C and D)**, mainly driven by the DMN_A and vLANG networks.

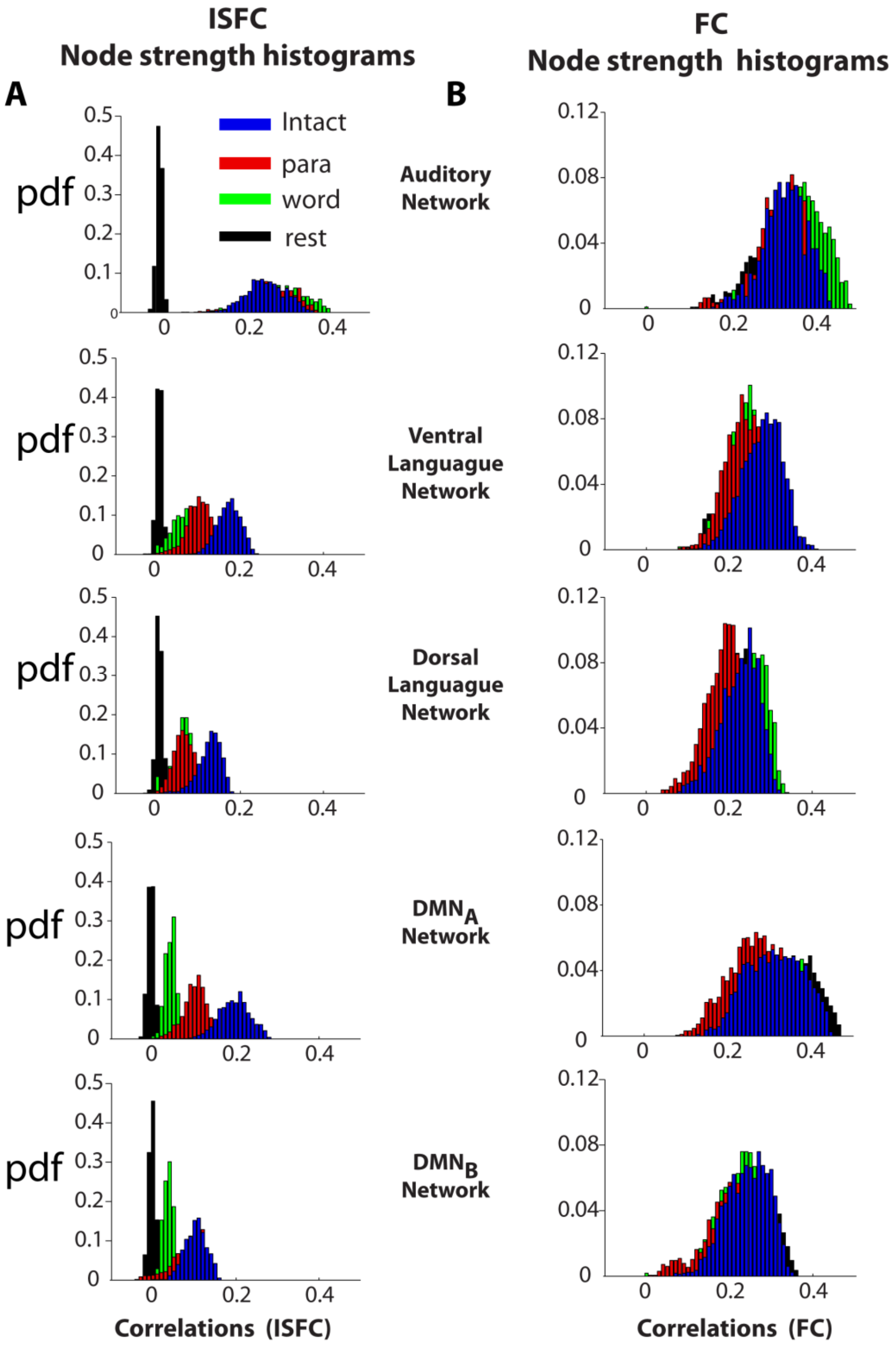

ISFC
Node strength histograms
FC
Node strength histograms
A
B
Intact
para
word
rest
pdf
Auditory Network
Ventral Language Network
Dorsal Language Network
DMN_A Network
DMN_B Network
Correlations (ISFC)
Correlations (FC)

Supplementary Figure 11 | Edge strength histograms of ISFC and FC for each network across four conditions. The node strength histogram shows the probability distribution function (pdf) of the mean of the correlations that each voxel maintains with all other voxels in the network.

(**A**) Separation of ISFC networks between temporally-scrambled versions of the story. The auditory network showed no selectivity to condition, the dorsal Language network was unable to distinguish the Paragraph Scramble from Word Scramble condition, and the MTL-based DMN network (DMN_B) did not differentiate between the Intact and Paragraph Scramble conditions. The most robust separation between conditions is demonstrated in the DMN_A and the ventral language networks. (**B**) When computed using standard FC, the pdfs overlap substantially across conditions for all networks.

Edge Number	Node – Node	Average FC during rest (n=18,r±SEM)
1	right IPL - right MFG	0.68±0.021
2	right IPL - right PREC	0.71±0.026
3	right IPL - right PREC	0.50±0.029
4	right IPL - right MTG	0.52±0.034
5	right IPL - left MTG	0.34±0.041
6	right IPL - left mPFC	0.40±0.036
7	right IPL - left PREC	0.63±0.027
8	right IPL - left MFG	0.51±0.031
9	right IPL - left IPL	0.66±0.027
10	right MFG - right PREC	0.55±0.019
11	right MFG - right PREC	0.46±0.035
12	right MFG - right MTG	0.40±0.040
13	right MFG - left MFG	0.27±0.040
14	right MFG - left mPFC	0.35±0.039
15	right MFG - left PREC	0.47±0.023
16	right MFG - left MFG	0.57±0.032
17	right MFG - left IPL	0.48±0.034
18	right PREC - right mPFC	0.40±0.031
19	right PREC - right MTG	0.42±0.033
20	right PREC - left MTG	0.28±0.039
21	right PREC - left mPFC	0.33±0.039
22	right PREC- left PREC	0.91±0.0073
23	right PREC- left MFG	0.38±0.0296
24	right PREC – left IPL	0.53±0.04
25	right mPFC - right MTG	0.60±0.029
26	right mPFC - left MTG	0.51±0.038
27	right mPFC - left MPFC	0.90±0.010
28	right mPFC - left PREC	0.42±0.03
29	right mPFC - left MFG	0.40±0.04
30	right mPFC - left IPL	0.43±0.04
31	right MTG - left MTG	0.56±0.036
32	right MTG - left mPFC	0.53±0.034
33	right MTG - left PREC	0.42±0.030
34	right MTG - left MFG	0.35±0.036
35	right MTG - left IPL	0.42±0.041
36	left MTG- left mPFC	0.54±0.040
37	left MTG - left PREC	0.33±0.037
38	left MTG - left MFG	0.36±0.031
39	left MTG - left IPL	0.40±0.032
40	left mPFC - left PREC	0.40±0.033
41	left mPFC - left MFG	0.45±0.036
42	left mPFC - left IPL	0.45±0.038
43	left PREC - left MFG	0.48±0.025
44	left PREC - left IPL	0.63±0.032
45	left MFG- left IPL	0.68±0.022

Supplementary Table 1: Default mode network edges, as defined using FC during rest. Related to Figure 3**.** Using average functional connectivity across 18 subjects during rest (seed region: PCC), we defined 10 DMN nodes across two hemispheres: IPL (Inferior Parietal Lobule), PREC/PCC (Precuneus/Posterior Cingulate Cortex), mPFC (medial prefrontal cortex), MFG (Middle frontal gyrus). The mean correlation ± SEM is presented for each edge.

Network	Region of Interest (Abbreviations)	Hemisphere	Number of Voxels (Group 1)	peak MNI coordinates (Group1) (mm)	Number of Voxels (Group 2)	peak MNI coordinates (Group2) (mm)	Regions
Auditory Network	A1+	right	254	53, -14, 4	329	54,-18,6	Primary Auditory Cortex
	A2	right	192	56, -23, 3	176	54,-23,-1	Secondary auditory cortex
	PCG	left	29	-48, -7, 47	30	-48,-7,46	Precentral gyrus
	A2	left	286	-55, -27, 2	294	-55,-29,-1	Secondary auditory cortex
	A1+	left	302	-50, -21, 6	360	-51,-22,6	Primary Auditory Cortex
Ventral Language network	TPJ	right	168	54, -50, 14	175	53,-50,21	Temporoparietal junction
	pSTS	right	37	55, -45, 11	103	56,-47,11	Superior temporal sulcus
	MTG	right	341	55, -31, -3	229	56,-33,-3	Medial temporal junction
	aSTS	right	64	57, -9, -18	104	55,-8,-18	Superior temporal sulcus
	aMTG	right	22	58, -6, -11	27	56,-9,-11	Medial temporal gyrus
	TP	right	88	51, 6, -25	56	50,9,-25	Temporal pole
	STG	right	19	33, 20, -18	24	34,20,-18	Superior temporal gyrus
	MFG	right	55	42, 11, 46	44	43,9,47	Middle frontal gyrus
	IFG	right	33	54, 26, 13	174	51,23,18	Inferior frontal gyrus
	SFG	right	321	8, 48, 33	339	8,45,35	Superior frontal gyrus
	SFG	left	427	-7, 48, 30	473	-7,46,31	Superior frontal gyrus
	IFG	left	5	-46,32,-8	4	-47,32,-10	Inferior frontal gyrus
	MFG	left	87	-40, 12, 45	102	-41,11,44	Middle frontal gyrus
	STG	left	13	-39, 18, -25	11	-39,18,-26	Superior temporal sulcus
	TP	left	45	-51, 3, -26	35	-51,3,-27	Temporal pole
	aMTG	left	100	-56, -14, -16	66	-56,-13,-18	Medial temporal gyrus
	aSTS	left	155	-54, -55, 14	66	-57,-50,11	Superior temporal sulcus
	MTG	left	177	-55, -39, -1	102	-56,-36,-8	Middle temporal gyrus
	pSTS	left	55	-49, -61, 19	93	-52,-54,18	Superior temporal sulcus
	TPJ	left	30	-51, -56, 31	88	-48,-60,21	Temporoparietal junction
Dorsal Language network	CN	right	15	11, 3, 9	10	11,3,8	Caudate nucleus
	IFG	right	401	47, 20, 12	215	45,19,6	Inferior frontal gyrus
	SFG	right	84	5, 23, 50	41	4,15,55	Superior frontal gyrus
	STG	right	176	57, -43, 20	84	58,-36,14	Superior temporal gyrus
	STG	left	211	-53, -50, 22	159	-55,-48,15	Superior temporal gyrus
	SFG	left	174	-4, 21, 49	129	-4,15,53	Superior frontal gyrus
	IFG	left	814	-46, 18, 12	791	-46,17,12	Inferior frontal gyrus
DMN_A	IPL	right	321	48, -60, 27	313	46,-62,28	inferior parietal lobule
	MFG	right	151	33, 24, 44	223	31,23,46	Middle frontal gyrus
	PREC/PCC	right	304	7, -57, 33	407	7,-55,31	Precuneus/Posterior Cingulate
	mPFC	right	42	6, 52, 6	77	6,53,5	medial Prefrontal cortex
	MTG	right	35	58, -12, -21	51	59,-10,-21	Middle temporal gyrus
	MTG	left	9	-57, -13, -22	16	-58,-11,-22	Middle temporal gyrus
	mPFC	left	31	-2, 54, -3	63	-2,54,-3	Medial prefrontal cortex
	PREC/PCC	left	498	-5, -57, 34	641	-6,-56,32	Precuneus
	MFG	left	166	-29, 20, 47	53	-28,22,46	Middle frontal gyrus
	IPL	left	339	-41, -68, 32	323	-41,-68,32	inferior parietal lobule
DMN_B (MTL Based)	PREC/PCC	right	443	8, -53, 30	276	8,-53,36	Precuneus/Posterior Cingulate
	IPL	right	263	40, -61, 43	262	42,-58,43	inferior parietal lobule/Angular Gyrus
	MFG	right	76	34, 35, 35	56	39,33,33	Middle frontal gyrus
	SFG	right	51	27, 19, 51	5	28,15,56	Superior frontal gyrus
	mPFC		92	0, 50, -9	53	-1,49,-10	Medial Prefrontal cortex
	SFG	left	56	-24, 18, 48	12	-22,13,54	Superior frontal gyrus
	MFG	left	13	-44, 32, 19	15	-45,30,27	Middle frontal gyrus
	IPL	left	176	-36, -59, 45	234	-37,-61,42	inferior parietal lobule/Angular Gyrus
	PREC/PCC	left	459	-6, -52, 28	292	-5,-50,33	Precuneus/Posterior Cingulate
	PHG	left	28	-29, -40, -17	28	-29,-40,-17	parahippocampal gyrus

Supplementary Table 2: 52 ROIs across five networks.

K-means clustering and local clustering (see methods) were applied to each one of the ISFC voxel-wise covariance matrices (n=18) during the intact story. 52 ROIs across five networks were defined for each one of the groups.

Supplementary Note 1: Statistical model and analytical results

1 Problem formulation

Suppose we have recorded neural data from *k* subjects. The neural signals X_i, measured from subject i, are in the form of a $p \times n$ matrix that contains signals from p neural sources over n time points. We consider a model of the form:

$$X_i = \left(A + D_i\right)S + I_i \qquad i = 1, \cdots, k \tag{1}$$

Our goal is to estimate the shared stimulus-induced covariance matrix, $C = AA^T$ given X_i for i=1…k.

For simplicity, we assume that the measured signals have unit variance. Each X_i has an intrinisc part I_i, which is stimulus independent, and a joint hidden variable S, that can be transformed within each subject by a subject-specific factor D_i. S is the stimulus-related joint set of $p \times n$ hidden variable elements in both columns and the rows, which are independent and identically distributed (i.i.d.) as $\mathcal{N}(0,1)$. The I_i are the $p \times n$ intrinsic components (including both spontaneous neural fluctuations and non-neural noise responses) with i.i.d. columns. The covariance of the columns of I_i is given by Q, which is common to all subjects. The unknown stimulus related covariance matrix is defined via the sum of a shared deterministic component (A) and a stimulus-induced individual random fluctuation term D_i whose elements are i.i.d. random variables distributed according to $\mathcal{N}(0, \sigma_D^2)$. We assume that D_i, S and I_i are all statistically independent.

2 Sample covariance

A first solution is to compute the sample covariances within a subject, as is done in standard functional connectivity (FC) analyses:

$$\hat{C}_i = \frac{1}{n} X_i X_i^T \tag{2}$$

Using the technical results below, for sufficiently large n and conditioned on D_i, this sample covariance converges to $(A+D_i)(A+D_i)^T+Q$. The error in using this sample covariance as an estimate of C reduces to

$$\mathrm{E}\left\{\left\|\hat{C}_i - C\right\|_{\text{fro}}^2\right\} \xrightarrow{n \gg p} \mathrm{E}\left\{\left\|D_i A^T + A D_i^T + D_i D_i^T + Q\right\|_{\text{fro}}^2\right\} \tag{3}$$

$$= (2p+2)\sigma_D^2 \left\|A\right\|_{\text{fro}}^2 + 2p\sigma_D^2 \mathrm{Tr}[Q] + \left\|Q\right\|_{\text{fro}}^2 + (2p^3+p^2)\sigma_D^4 \tag{4}$$

where || ||fro is the Frobenius norm. This error does not decrease by increasing n or k. It reaches an error floor which is dominated by the statistics of the individual fluctuations D_i (σ_D^2) and the intrinsic component I_i (Q). In addition, averaging over subjects does not eliminate Q the influence of Q. For simulation results see Supplemental Figure 3A.

3 Sample cross covariance

A second approach is to compute the sample cross covariances between single subject responses and the average of the other subjects in the group. This approach eliminates the intrinsic components, and some of the stimulus-induced individual fluctuations. Specifically, we define the i th estimate as the subject-based Inter-subject functional correlation (ISFC) :

$$\hat{C}_{i:} = \frac{1}{n} X_i \left[\frac{1}{k-1}\sum_{j\neq i} X_j^T\right] \tag{5}$$

For sufficiently large n and conditioned on the D_i's, this average cross covariance converges to

$$(A+D_i)\left[\frac{1}{k-1}\sum_{j\neq i}(A+D_j)^T\right] \tag{6}$$

The main advantage is that the individual covariance Q disappears, because I_i and I_j are independent for $i \neq j$. Thus,

$$\mathrm{E}\left\{\left\|\hat{C}_{i:} - C\right\|_{\text{fro}}^2\right\} \xrightarrow{n \gg p} \mathrm{E}\left\{\left\|D_i A^T + A\left[\frac{1}{k-1}\sum_{j\neq i} D_j^T\right] + D_i\left[\frac{1}{k-1}\sum_{j\neq i} D_j^T\right]\right\|_{\text{fro}}^2\right\} \tag{7}$$

$$= p\sigma_D^2 \|A\|_{\text{fro}}^2 + p\frac{\sigma_D^2}{k-1}\|A\|_{\text{fro}}^2 + p^3\sigma_D^2 \frac{\sigma_D^2}{k-1} \tag{8}$$

It is clear that the error in this case is much smaller than for the sample covariance computed in Section 2 above. Here, it no longer depends on Q, and the last two terms decrease with $k-1$. Nonetheless, we still have a positive error floor, because the first term is independent of k. We used the subject-based ISFC matrices for classification in Figures 3,4,7. For simulation results see Supplemental Figure 3B.

4 Averaged sample cross covariances

In order to reduce the error floor to zero, we take another average with respect to i . This leads to the following estimate, which is called "group-based ISFC":

$$\hat{C} = \frac{1}{k}\sum_i \hat{C}_{i:} \tag{9}$$

$$= \frac{1}{n}\frac{1}{k(k-1)}\sum_{i,j:i\neq j} X_i X_j^T \tag{10}$$

As before, for sufficiently large n and conditioned on the D_i's, this average cross covariance converges to

$$\frac{1}{k(k-1)}\sum_{i,j:i\neq j}(A+D_i)(A+D_j)^T \tag{11}$$

Thus,

$$\mathrm{E}\left\{\left\|\hat{C}-C\right\|_{\text{fro}}^2\right\} \overset{n\gg p}{\rightarrow} \mathrm{E}\left\{\left\|\frac{1}{k}\sum_i D_i A^T + A\left[\frac{1}{k-1}\sum_{j\neq i} D_j^T\right] + \frac{1}{k(k-1)}\sum_{i,j:i\neq j} D_i D_j^T\right\|_{\text{fro}}^2\right\} \tag{12}$$

$$= p\frac{\sigma_D^2}{k}\|A\|_{\text{fro}}^2 + p\frac{\sigma_D^2}{k-1}\|A\|_{\text{fro}}^2 + p^3\frac{\sigma_D^2}{k}\frac{\sigma_D^2}{k-1} \overset{k\gg p^3}{\rightarrow} 0 \tag{13}$$

Due to the additional averaging over i, all the terms decrease by another factor of k. For sufficiently large k (in comparison to the dimension p^3) the error will converge to zero. For numerical simulation results indicating the error in typical neuroimaging settings, see Supplemental Figure 3C.

5 Technical results

$$SS^T / n \xrightarrow{n \gg p} I \tag{14}$$

$$I_i I_j^T / n \xrightarrow{n \gg p} 0 \tag{15}$$

$$\left[\frac{1}{k-1} \sum_{j \neq i} D_j^T \right] \sim \mathcal{N}_{\text{i.i.d.}} \left(0, \frac{\sigma_D^2}{k-1} \right) \tag{16}$$

$$\mathrm{E}\left\{ \left\| DA^T \right\|_{\text{fro}}^2 \right\} = p\sigma_D^2 \left\| A \right\|_{\text{fro}}^2 \tag{17}$$

$$\mathrm{E}\left\{ \mathrm{Tr}\left\{ DA^T DA^T \right\} \right\} = \sigma_D^2 \left\| A \right\|_{\text{fro}}^2 \tag{18}$$

$$\mathrm{E}\left\{ \mathrm{Tr}\left\{ DQD^T \right\} \right\} = p\sigma_D^2 \mathrm{Tr}\left\{ Q \right\} \tag{19}$$

$$\mathrm{E}\left\{ \left\| DD^T \right\|_{\text{fro}}^2 \right\} = \left(2p^3 + p^2 \right) \sigma_D^4 \tag{20}$$

$$\mathrm{E}\left\{ \left\| D_i D_j^T \right\|_{\text{fro}}^2 \right\} = p^3 \sigma_D^4 \tag{21}$$

$$\mathrm{E}\left\{ \left\| A \left[\frac{1}{n} \mathrm{Wishart}(p) - I \right] B^T \right\|_{\text{fro}}^2 \right\} = \frac{\left\| AB^T \right\|_{\text{fro}}^2 + \left\| A \right\|_{\text{fro}}^2 \left\| B \right\|_{\text{fro}}^2}{n} \tag{22}$$

Supplementary Note 2 : Across-subjects classification of ISFC patterns vs. FC patterns

ISFC classification of condition: For each condition (Rest, Word Scramble, Paragraph Scramble, Intact Story) we had data from M = 18 subjects. Correlation matrices were computed over the entire timecourse (280 TRs for 'Pie Man') between all pairs of the 10 DMN nodes, leading to a 10 x 10 correlation matrix. On each iteration of the classification algorithm, we left out four subjects, one from each of the N = 4 conditions, to be used as test subjects. For each of the N = 4 conditions separately, we computed an ISFC fingerprint, C_n using the M-1 subjects remaining in that condition. This resulted in a set of N = 4 ISFC fingerprint templates (C_n, n=1, 2…N; N = 4), one for each condition. The ISFC fingerprints were then used for classification of the four held-out subjects, according to Equation 1 below. For each held-out test subject, *s*, and for each condition, *n*, (one in each of the 4 conditions), we calculated an ISFC matrix between that subject and the average data from the 17 (M-1) subjects remaining in each condition to produce a matrix $C_{s,n}$ ($C_{s,n}$, s=1, 2…N; n = 1,2, …N; N = 4). Our predicted condition for the held-out subject, s, was the condition *n* that maximized the Pearson correlation between C_n and $C_{s,n}$, as given by:

$$\hat{n}_s = \underset{n=1:N}{\arg\max} \left\{ Corr(c_{s,n}, c_n) \right\} \quad (1)$$

Classification accuracy was then computed as the proportion of times a subject was assigned to the correct condition.

FC classification of condition: we used the same ISFC procedure as described above. The only difference was that given a test subject, s, we calculated the correlation matrix of that condition (i.e., FC) within that subject. Hence, for a given test subject, we have *one* FC correlation matrix C_s (in contrast to ISFC, where we have 4 matrices). Our predicted condition is the condition n that maximizes the correlation between C_s and C_n. The matrix C_n was calculated in the training phase using within-subject FC and was then averaged across the M-1 subjects from that condition. Then the best matching condition was computed as:

$$\hat{n}_s = \underset{n=1:N}{\arg\max} \left\{ Corr(c_s, c_n) \right\} \quad (2)$$

ISFC classification of non-overlapping intervals: We used the leave-one-out procedure among the M=18 subjects that listened to the intact condition. Within each of the 10 DMN regions, we divided the timecourses (length 280 TRs) into 14 non-overlapping intervals of 20TRs each. For each interval we calculated the ISFC correlation matrix (10 x 10 ROIs) over M-1 subjects, and used them to predict the interval index, *n*, (out of 14) of the left-out subject. We used the same procedure as with *ISFC classification of condition*, as reflected by equation 5. For the movie "Twilight Zone", we divided the BOLD time series into 16 to non-overlapping intervals of 60TRs each.

FC classification of non-overlapping intervals: The procedure is same as for *ISFC classification of non-overlapping intervals,* except that only one correlation matrix is calculated per interval per leave-one subject (compared to the 14 matrices calculated using ISFC), as reflected in Equation 2.

Supplemental References

1 Chang, C., Cunningham, J. P. & Glover, G. H. Influence of heart rate on the BOLD signal: the cardiac response function. *NeuroImage* **44**, 857-869, doi:10.1016/j.neuroimage.2008.09.029 (2009).

2 Shmueli, K. *et al.* Low-frequency fluctuations in the cardiac rate as a source of variance in the resting-state fMRI BOLD signal. *Neuroimage* **38**, 306-320, doi:S1053-8119(07)00670-2 [pii]10.1016/j.neuroimage.2007.07.037 (2007).

3 Chang, C. & Glover, G. H. Relationship between respiration, end-tidal CO2, and BOLD signals in resting-state fMRI. *NeuroImage* **47**, 1381-1393, doi:10.1016/j.neuroimage.2009.04.048 (2009).

4 Power, J. D., Barnes, K. a., Snyder, A. Z., Schlaggar, B. L. & Petersen, S. E. Spurious but systematic correlations in functional connectivity MRI networks arise from subject motion. *NeuroImage* **59**, 2142-2154, doi:10.1016/j.neuroimage.2011.10.018 (2012).

5 Chang, C. & Glover, G. H. Effects of model-based physiological noise correction on default mode network anti-correlations and correlations. *NeuroImage* **47**, 1448-1459, doi:10.1016/j.neuroimage.2009.05.012 (2009).

6 Dodell-Feder, D., Koster-Hale, J., Bedny, M. & Saxe, R. fMRI item analysis in a theory of mind task. *Neuroimage* **55**, 705-712, doi:10.1016/j.neuroimage.2010.12.040 (2011).

7 Chen, J. *et al.* Accessing Real-Life Episodic Information from Minutes versus Hours Earlier Modulates Hippocampal and High-Order Cortical Dynamics. *Cereb Cortex*, doi:10.1093/cercor/bhv155 (2015).

8 Buckner, R. L., Andrews-Hanna, J. R. & Schacter, D. L. The brain's default network: anatomy, function, and relevance to disease. *Annals of the New York Academy of Sciences* **1124**, 1-38, doi:10.1196/annals.1440.011 (2008).

9 Andrews-Hanna, J. R., Reidler, J. S., Sepulcre, J., Poulin, R. & Buckner, R. L. Functional-anatomic fractionation of the brain's default network. *Neuron* **65**, 550-562, doi:10.1016/j.neuron.2010.02.005 (2010).

10 Hickok, G. & Poeppel, D. The cortical organization of speech processing. *Nat Rev Neurosci* **8**, 393-402, doi:nrn2113 [pii]10.1038/nrn2113 (2007).

11 Poeppel, D. & Hickok, G. Towards a new functional anatomy of language. *Cognition* **92**, 1-12, doi:S0010027703002257 [pii]10.1016/j.cognition.2003.11.001 (2004).

12 Lloyd, S. Least squares quantization in PCM. *IEEE Transactions on Information Theory* **28**, 129-137, doi:10.1109/TIT.1982.1056489 (1982).
